# An Evolutionary Perspective of the Lipocalin Protein Family

**DOI:** 10.3389/fphys.2021.718983

**Published:** 2021-08-23

**Authors:** Sergio Diez-Hermano, Maria D. Ganfornina, Arne Skerra, Gabriel Gutiérrez, Diego Sanchez

**Affiliations:** ^1^Departamento de Bioquimica y Biologia Moleculary Fisiologia, Instituto de Biologia y Genetica Molecular, Universidad de Valladolid-Consejo Superior de Investigaciones Cientificas (CSIC), Valladolid, Spain; ^2^Lehrstuhl für Biologische Chemie, Technische Universität München, Freising, Germany; ^3^Departamento de Genética, Universidad de Sevilla, Seville, Spain

**Keywords:** Lipocalin, Calycin, protein phylogeny, functional divergence, molecular evolution

## Abstract

The protein family of Lipocalins is ubiquitously present throughout the tree of life, with the exception of the phylum Archaea. Phylogenetic relationships of chordate Lipocalins have been proposed in the past based on protein sequence similarities, but their highly divergent primary structures and a shortage of experimental annotations in genome projects have precluded a well-supported hypothesis for their evolution. In this work we propose a novel topology for the phylogenetic tree of chordate Lipocalins, inferred from multiple amino acid sequence alignments. Sixteen jawed vertebrates with fair coverage by genomic sequencing were compared. The selected species span an evolutionary range of ∼400 million years, allowing for a balanced representation of all major vertebrate clades. A consensus phylogenetic tree is proposed following a comparison of sequence-based maximum-likelihood trees and protein structure dendrograms. This new phylogeny suggests an APOD-like common ancestor in early chordates, which gave rise, via whole-genome or tandem duplications, to the six Lipocalins currently present in fish (APOD, RBP4, PTGDS, AMBP, C8G, and APOM). Further gene duplications of APOM and PTGDS resulted in the altogether 15 Lipocalins found in contemporary mammals. Insights into the functional impact of relevant amino acid residues in early diverging Lipocalins are also discussed. These results should foster the experimental exploration of novel functions alongside the identification of new members of the Lipocalin family.

## Introduction

Lipocalins form an ancestral protein family so far found in all kingdoms of life except for Archaea. The evolutionary paths followed by the Lipocalins have been explored using standard *in silico* molecular evolution methodologies based on protein sequence alignments, exon-intron architecture and protein tertiary structure comparisons (Ganfornina et al., 2000; Gutierrez et al., 2000; Sanchez et al., 2003, 2006; Lakshmi et al., 2015). These reports supported an evolutionary hypothesis where one (or a couple) of chordate Lipocalin genes found in separate chromosomes (e.g., APOD and RBP4 in human chromosomes 3 and 10 respectively) would repeatedly duplicate to achieve the remaining 15–20 Lipocalins that would cluster in a chromosome (e.g., human chromosome 9). See Charkoftaki et al. (2015) for a detailed chromosomal location of human and mouse genes.

Previous reports aimed at maximizing the number of protein sequences to be included, which might bias phylogenetic relationships in tree nodes overrepresented in databases due to the preferential sequencing of model organisms or to genome projects that have been prioritized. Some reports focused on pairwise comparisons of two organisms (Charkoftaki et al., 2015), which hinders a complete phylogenetic interpretation of the family. Other works (Lakshmi et al., 2015) studied a reduced number of Lipocalins, yet without a clear selection motive, in the context of other structurally related protein families.

In this work we focus on chordate Lipocalins, the central theme of this Topic Series. To identify the proteins to be included in the analysis, we selected comparable numbers of organisms belonging to representative vertebrate classes, whose genomes were sequenced with good coverage. We then aligned the recovered Lipocalin amino acid sequences and built phylogenetic trees following a maximum-likelihood reconstruction method. Furthermore, we compared the protein structure space of human Lipocalins and its dendrogram with the sequence-based phylogenetic tree.

Finally, we identified particular residues accounting for both the divergence and the specificities of the main Lipocalin clades that appeared early in chordate evolution. Marking such residues as important will be useful to reveal protein regions relevant for known or novel functions that can be further experimentally tested. With these results we propose an evolutionary history for Lipocalins in chordates.

## Methods

**FIGURE 1:**
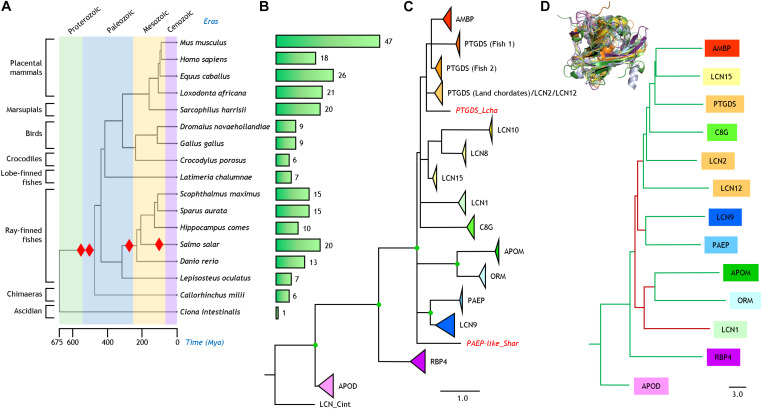
Phylogenetic relationships of Lipocalins**. (A)** Phylogeny and evolution timescale of the organisms selected for our analysis. Red diamonds indicate whole genome duplications. **(B)** Bars depicting the number of *bona fide* Lipocalins found in each organism. **(C)** Phylogenetic tree of 250 Lipocalin protein sequences reconstructed from a structure-based multiple sequence alignment and a ML method. The tree was rooted with a tunicate Lipocalin (LCN_Cint) considered as outgroup. Unsupported nodes (bootstrap <65%) are excluded and branches joined as a polytomy. Lipocalin monophyletic clades (bootstrap >85%) are shown collapsed and color-coded. Scale branch length represents number of amino acid substitutions per site. Individual Lipocalins with unsupported groupings are shown in red and italics. Green dots point to full bootstrap support. **(D)** Dendrogram depicting the relationships of thirteen human Lipocalins structurally aligned (3D superimposition shown as an inset). Scale branch length represents distances obtained from the DALI similarity matrix. Branches in green and maroon point to relationships concordant or discordant, respectively, with the protein sequence-based phylogeny in **(C)**.

Sixteen jawed vertebrates (gnathostomata) were selected for analysis (Figure 1A). These species cover vertebrate clades that have evolved during the last ∼400 My. Their genome sequencing projects show fair coverage and annotation. A tunicate (*Ciona intestinalis*) was also used to find Lipocalins that could serve as outgroup for our phylogenetic trees. A Lipocalin protein sequence search was performed using PSI-BLAST (Altschul et al., 1997), starting with the set of eighteen human Lipocalin sequences used in our previous phylogenetic studies (Sanchez et al., 2006). We then searched each protein against the genome assemblies of the selected organisms (Supplementary File 1 and Supplementary Table 1).

The recovered sequences were checked for full transcript coverage by comparison with the RefSeq_RNA database. The protein sequence of the Lipocalin Alpha-1-microglobulin was selected from the Ambp precursor protein, genetically encoded as a fusion protein with Bikunin (a non-Lipocalin trypsin inhibitor). The final list of proteins considered for our analysis is shown in Supplementary File 1 (Supplementary Table 2). Sequences are named with an abbreviated species name and a Lipocalin label based on the human genome nomenclature.

Prediction of N-terminal signal peptides, present in most Lipocalins, was performed with SignalP-5.0 (Almagro Armenteros et al., 2019), and the predicted fragment was removed from sequences entering the analysis. Multiple sequence alignments (MSA) of Lipocalin mature protein sequences were then carried out using MAFFT v7.475 (Katoh and Standley, 2013) according to the following workflows: (1) To study the general phylogeny of chordate Lipocalins, we first performed a MSA with the iterative refinement G-INS-I method for the terrestrial early diverging (ED) Lipocalins. We then used the MAFFT-Add program to include the remaining terrestrial and fish Lipocalins with a progressive G-INS-I method keeping alignment length (Parameters: BLOSUM62; Gap 1.53; Offset 0.0). (2) To study within-clade phylogenies we used the MAFFT G-INS-I method with structural masks based on the human resolved tertiary structures. (3) For functional divergence analyses, we selected five ED-Lipocalins (APOD, RBP4, PTGDS, AMBP and C8G) and generated MSAs for clade pairs (each clade with the most related one in the general tree) using iterative G-INS-I MAFFT. For each MSA, we used a structural mask as above. All MSAs generated in our analyses appear in Supplementary File 2.

Phylogenetic trees were inferred following a maximum-likelihood (ML) method with IQ-TREE (Nguyen et al., 2015). The automatic ModelFinder selected JTT + F + R5 as the best model for the general tree, taking into account rate heterogeneity (Kalyaanamoorthy et al., 2017). An Ultrafast Bootstrap approximation with 1,000 replicas was used to estimate nodes support (Hoang et al., 2018). The same automatic approach of ModelFinder considering rate heterogeneity was used for individual clade phylogenies. The tree files generated in this work are available in Supplementary File 2. Tree visualization and drawing were performed with FigTree v 1.4.4^1^ and iTOL^2^.

Functional divergence of paralogous proteins and their correlated functional specificity, were studied with DIVERGE 3.0 (Gu et al., 2013) and JDet 1.4.9 (Muth et al., 2012) using the MSAs for clade pairs. This approach compares each Lipocalin with the expected evolved duplicate according to the ML general family tree. We followed the suggested DIVERGE protocol and selected residues with a false discovery rate (FDR) limit of 5% and supported by the Site-Specific Posterior Profile (SSPP) Probability. The Type-I divergence analysis files and the summary Table presenting the statistical results for the comparisons in each pair of Lipocalin clades are shown in Supplementary File 3. When using JDet to highlight specificity-determining positions (SDPs), we selected residues with a threshold of XDet:0.6, Entropy:2.5 and S3:10.0. The pairwise MSA used by JDet and its output are shown in Supplementary File 4. The 3D structures of relevant Lipocalins including selected residues were depicted with ViewerLite 4.2 or PyMOL 2.3.3.

## Results and Discussion

The phylogenetic relationship and evolutionary timescale of the sixteen jawed vertebrates used in our study were estimated with TimeTree (Kumar et al., 2017; Figure 1A). Red diamonds point at whole genome duplications (WGD) currently proposed to have occurred in chordates (Meyer and Van de Peer, 2005; Kasahara, 2007). Figure 1B shows the number of Lipocalins recovered for each species with our protein sequence similarity search in each genome. From the single Lipocalin detected in the urochordate *Ciona*, the graph helps to visualize the step-wise expansion of this protein family in early marine and land vertebrates due to WGD (leading to 6–7 Lipocalins), as well as new rounds of WGD in fish (resulting in sets of 10–20 Lipocalins). Further increases in the number of mammalian Lipocalins, some of them quite substantial, can be explained by tandem gene duplications, as it has been reported for other gene families (Gaunt, 2015).

### Protein Sequence-Based Lipocalin Evolution

Our similarity search in the selected sixteen vertebrate genomes rendered 249 genes. After sequence processing, MSA and ML phylogenetic reconstruction, the resulting tree is shown in Figure 1C rooted with the *Ciona* Lipocalin. The nodes appearing in the tree are supported by bootstrap values >60%. Individual Lipocalin clades were supported by values >85% and are shown collapsed in the tree with a triangle whose area is proportional to the number of genes monophyletically related. Nodes with full 100% support are highlighted as green dots. Although we initially used the gene names present in the genome databases, we here propose to standardize Lipocalin names based on the strong support of our phylogenies, using the human genome nomenclature as a reference, combined with number-letter suffixes (Supplementary Table 2 and Supplementary File 1). In this sense, the long-studied odorant-binding Lipocalins fully group together with LCN1 and are correspondingly renamed in our phylogeny as such for all species used in this work. Similarly, the rodent urinary proteins fall into the LCN9 monophyletic clade. An intriguing case is PTGDS, which is distributed over three independent and highly supported (>95%) clades. Two of these clades are composed of fish genes, while one combines in a single clade the terrestrial vertebrate PTGDS, LCN2, and LCN12.

The reconstructed phylogeny reinforces the hypothesis that APOD and RBP4 are the earliest diverging among ED Lipocalins. All other extant Lipocalins in chordates join in a monophyletic group. Within that group our organism-based phylogeny resolves several relationships that were not firmly supported in previous phylogenies. LCN9 relates to PAEP, and APOM relates to ORM. These links were previously suggested in a mouse-human Lipocalin comparison (Charkoftaki et al., 2015), but without evidence of phylogenetic support. Also, AMBP groups monophyletically with the two fish PTGDS clades. Finally, a different set of Lipocalins (LCN8, LCN10 and LCN15) forms another cluster with strong support.

### Protein Structure-Based Lipocalin Relationships

Although the number of resolved tertiary structures for vertebrate Lipocalin is still insufficient, we used the DALI server (Holm, 2020) to structurally align thirteen human Lipocalins (3D superimposition shown in Figure 1D). The dendrogram produced by DALI, based on average linkage clustering of the Z-scores of the structural similarity matrix, reasonably matches the topology of our amino acid sequence-based phylogeny (tree branches labeled in green in Figure 1D). Only LCN1 relates structurally to APOM and ORM in a different tree location (brown branches in Figure 1D). These structural relationships coming from a different comparison methodology, but lacking an evolutionary perspective, endorse the Lipocalin clade associations reported above.

### Protein Sequence-Based Phylogenies and Organismal Representation of Lipocalin Clades

**FIGURE 2:**
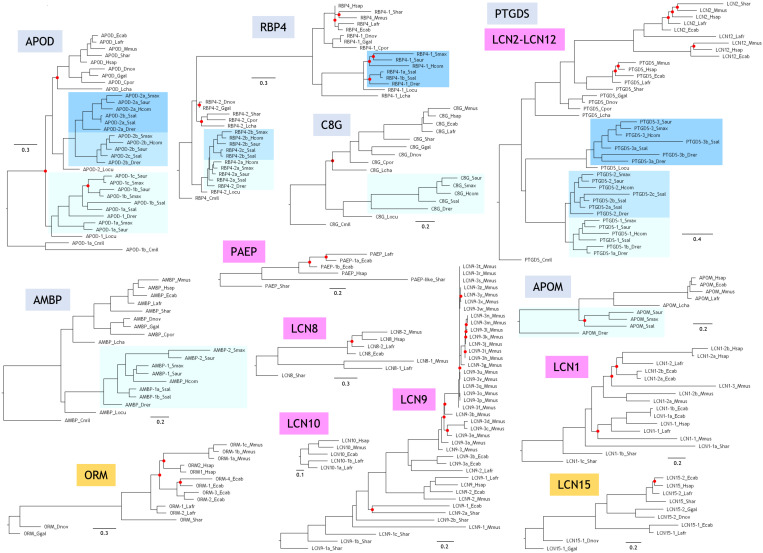
Phylogenetic trees of Lipocalin clades. Each tree is based on structure-based multiple protein sequence alignments and a ML method. Trees were rooted with the Lipocalin(s) of the earliest diverging organism as shown in Figure 1A. Unsupported nodes (bootstrap <65%) are marked by a red dot. Scale branch lengths represent the number of amino acid substitutions per site.

Phylogenetic relationships of independent Lipocalin clades are shown in Figure 2, each rooted with the protein(s) found in the organism showing the earliest time of divergence in Figure 1A. The six Lipocalin clades showing fish representatives (names boxed in light blue) are the ones defined as ED. Two clades debut in birds (names boxed in orange), while seven additional Lipocalins are latecomers, appearing during mammalian evolution (names boxed in purple). Except for APOM, all ED Lipocalins are present in cartilaginous fish, ancient ray-finned fish, and modern fish. Interestingly, APOD, RBP4, and PTGDS show groups of paralogous genes (boxed in shades of blue) reflecting the combination of regional tandem gene duplications with the different rounds of WGD that occurred in fish. However, AMBP, C8G, and APOM only show a single monophyletic fish group, suggesting processes of gene loss after genome duplication particularly prominent for APOM.

Two novel Lipocalins (ORM and LCN15) appear in our sample of terrestrial vertebrates. However, they are missing in the crocodile *C. porosus*, as it also happens with APOM, suggesting specific gene losses in this reptile representative.

Mammals display a great expansion of Lipocalins, mostly due to numerous and subsequent tandem gene duplications. As a result, five novel Lipocalins (PAEP, LCN1, LCN2, LCN8, and LCN9) are found in the marsupial *S. harrisii*, some of them showing evidence of recent gene duplications. Finally, our sample of placental mammals incorporate two novel Lipocalins (LCN10 and LCN12), configuring the final fifteen *bona fide* extant Lipocalins revealed by our species selection. This set is clearly the result of gene duplication and gene loss processes, with interesting selective losses resulting in Lipocalin pseudogenes still recognizable in late diverging (LD) Lipocalins (Schiefner et al., 2015). Our genome-wide strategy for Lipocalin identification does not retrieve some proteins considered Lipocalins in other published reports. To cite just an example, a mouse ortholog of LCN15 is missing in our sequence list, while it is reported as a mouse Lipocalin by Charkoftaki et al. (2015). Our inclusion criteria do not include fatty acid-binding proteins as genuine Lipocalins, as it is the case for the alleged mouse Lcn15 homolog. They form a different protein family within the Calycin superfamily (Sanchez et al., 2006). We must therefore pay attention to these incorrect family assignments while further data help to curate genome annotations.

### Functional Divergence Along the Evolution of Terrestrial ED Lipocalins

**FIGURE 3:**
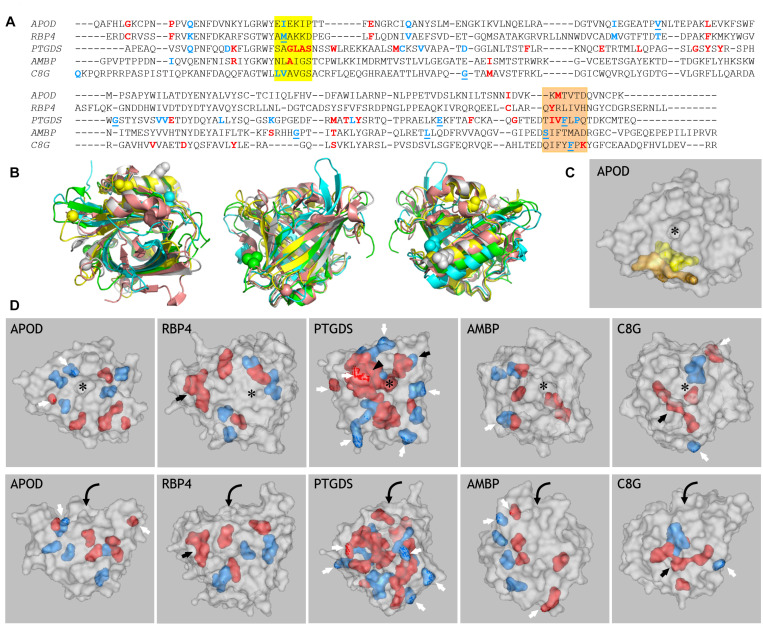
Prediction of amino acid residues related to functional divergence in ED Lipocalins. **(A)** MSA of the five human representatives of the ED Lipocalins selected for this analysis. Residues related to Type-I divergence are labeled in blue, with underlined residues showing SSPP-probability support. Specificity-determining positions (SDPs) are labeled in red. Colored boxes mark two sequence regions enriched in functionally divergent residues. **(B)** Human Lipocalin structures superimposed, based on the structurally conserved beta-barrel, and colored (APOD, green; RBP4, cyan; PTGDS, gray; AMBP, yellow; C8G, salmon). Views into the ligand pocket, at the front side and the back side, revealing the alpha-helix attached to the beta-barrel, are shown. Colored spheres display SSPP-supported Type-I residues in all five Lipocalins. **(C)** Space-filled view of ApoD highlighting the residues marked by the two colored boxes in **(A)**. **(D)** Gray-colored space-filled views of the five human Lipocalin structures showing the color-coded residues identified in **(A)**. Panels in the upper row show views into the Lipocalin hydrophobic pocket (asterisk) while panels in the lower row show side views of the beta-barrel. Residues accessible at the protein surface (white arrows), or buried within the structure (black arrows) are pointed. Arrowhead points to a side wall of the binding pocket in PTGDS, and curved arrows suggest the binding pocket entrance on the side views of Lipocalins.

In an attempt to elucidate the functional traits and constraints potentially conditioning Lipocalin evolution in chordates, we have analyzed MSAs for clade pairs of the Lipocalins APOD, RBP4, PTGDS, AMBP, and C8G using the terrestrial vertebrate sequences. These pairs were explored following the predicted pattern of successive gene duplications (Supplementary File 3). The analysis identifies residues that experience altered functional constraints, as positions in the alignment that are variable in one clade and conserved in the other (Gu et al., 2013) or that define functional specificities as positions that are conserved but different in each clade (Muth et al., 2012). Figure 3A shows these predicted residues, colored in blue (Type-I) or red (SDPs), in the context of a MSA of the five human representatives of the Lipocalins mentioned above. To analyze the positions in the Lipocalins tertiary structure of residues showing correlated changes in evolutionary rates within a clade (Type-I), we focused on those best supported by SSPP-probability (underlined in Figure 3A) and displayed them as spheres in the superimposed Lipocalin structures (Figure 3B). The highlighted residues tend to group in the three-dimensional structure, mostly on the surface of the Lipocalins and less in the ligand pockets. This result highlights the importance of residues potentially involved in protein-protein interactions in the radiation of Lipocalins, while those playing a role in ligand specificity might contribute less to the site-specific rate of change between Lipocalins.

When considering amino acids potentially involved in functional divergence (Type I and SDP), a group of them concentrate in two interesting areas in the MSA in all five Lipocalins (boxed in yellow and orange in Figure 3A). These regions therefore behave as hot-spots for generation of functional novelty (Ganfornina and Sánchez, 1999) along Lipocalin evolution. When located in a 3D structure (APOD shown as example in Figure 3C), the two regions pack together, with many of their residues lining the protein surface further highlighting intermolecular interactions as a relevant driving force.

We then explored the residues selected by DIVERGE (Type-I) and JDet (SDP) for each Lipocalin clade pair, and labeled them with different colors on the surface of each human Lipocalin structure (Figure 3D). A striking result of these studies is the large number of residues predicted to sustain the functional divergence of PTGDS from RBP4, with many residues located on the protein surface (white arrows) and a compact patch of residues (black arrowhead) lining a side wall of the binding pocket (asterisk). It is also interesting that the catalytic cysteine residue responsible for the unique enzymatic activity of PTGDS (black arrow) appears as a site-specific functional divergence, thus validating the methodology used. That PTGDS appears highly prone to shifted functional constraints might underlie its peculiar polyphyletic topology in the general ML tree discussed in section “Protein Sequence-Based Lipocalin Evolution” (Figure 1B), and could also represent the source of sequence variation that fueled the radiation of late-diverging Lipocalins from an ancestral PTGDS.

In the other clade pair analyses, a smaller number of residues are predicted to relate to functional divergence. These residues arrange around the binding pocket in APOD, with few of them accessible at the protein surface (white arrows). A similar pattern is seen for RBP4 in addition to two cysteine residues forming a peculiar third disulfide bond (a distinct feature of RBP4) that were correctly selected as SDPs (black arrow). AMBP shows an interesting polarization in the positioning of residues potentially contributing to functional divergence (easily seen in the side view of the molecule, bottom panel). Two of the selected amino acids of C8G contribute to the protein surface (white arrows), while three hydrophobic residues line up in the inner part of the protein (black arrow).

### An Evolutionary Perspective of the Lipocalin Protein Family

**FIGURE 4:**
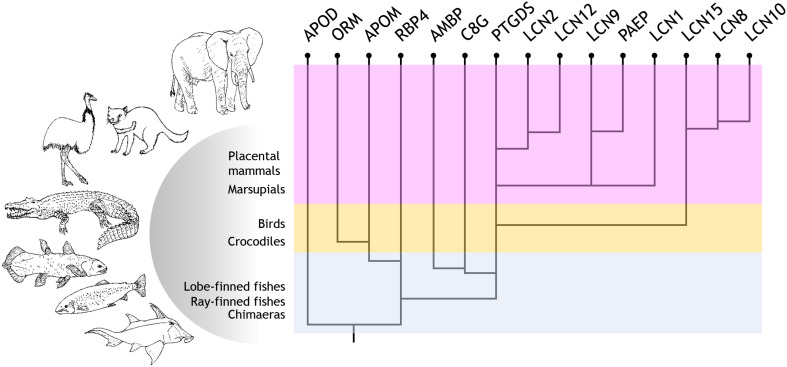
Current proposal for the evolutionary history of Lipocalin in chordates. Evolutionary path suggested for Lipocalin duplication and diversification in chordates. Six Lipocalins evolved during fish evolution, while at least nine novel Lipocalins evolved in terrestrial vertebrates.

Parsing our results, we propose an evolutionary path for Lipocalin duplication and diversification in chordates, which is schematically depicted in Figure 4. Starting from an APOD-like common ancestor, early chordates start a series of WGD and tandem duplications that soon gave rise to six Lipocalins present in extant fish (APOD, RBP4, PTGDS, AMBP, C8G, and APOM). Based on the topologies of our new phylogenetic trees, our previously studied Lipocalin gene structure similarities, their three-dimensional structures, their diverse posttranslational features and protein expression patterns, as well as their organismal representation (Ganfornina et al., 2000, 2006; Gutierrez et al., 2000; Sanchez et al., 2003, 2006; Schiefner and Skerra, 2015), we propose the following evolutionary scenario for the subsequent history leading to the extant vertebrate Lipocalin set. An ancestral RBP4 duplicated and diverged into PTGDS. PTGDS, with its tendency to sequence divergence, underwent an early tandem duplication that gave rise to C8G and AMBP, two Lipocalins that share a particular exon-intron gene structure unique in the family. Whether it is C8G or AMBP the first to arise early in chordates is debatable. However, AMBP appears as a fusion between the Lipocalin Alpha-1-microglobulin and the proteinase inhibitor Bikunin. This lead us to propose a more parsimonious hypothesis with C8G being the descendant of PTGDS, and a subsequent gene duplication in conjunction with a gene fusion event generating AMBP. RBP4 also gave rise to APOM, with which it shares a unique pattern of three disulfide bridges (Schiefner and Skerra, 2015), and APOM in turn could have generated ORM in terrestrial vertebrates. Three independent PTGDS gene duplications in birds, early mammals and placental mammals, as well as local gene duplications in several clades, account for the catalog of Lipocalins present in contemporary mammals.

In summary, a protein sequence-based phylogeny of selected organisms supports a novel tree topology of chordate Lipocalins, which in turn enables us to propose an upgraded hypothesis for their evolutionary history. Moreover, we report a number of amino acid residues related to the functional divergence of early diverging Lipocalins. Needless to say that the hypothetical evolutionary path we offer for Lipocalins will need to be revisited whenever new vertebrate genomes become fully annotated. Inclusion of Lipocalins from other phyla as well as of sister families that together compose the Calycin superfamily, would further contribute to a comprehensive view of the evolution of these ancient and widespread genes. Finally, the *in silico* method used here to identify residues undergoing rapid divergence or functional specification in particular Lipocalins proved to be a helpful approach for the design of future experiments aiming at defining the origin of the diverse physiological roles of these essential proteins.

## Data Availability Statement

The original contributions presented in the study are included in the article/Supplementary Material, further inquiries can be directed to the corresponding author/s.

## Author Contributions

GG, MG, and DS: conceptualization. SD-H, GG, AS, and DS: bioinformatics analyses. DS: writing—original draft. SD-H, MG, GG, AS, and DS: writing—review and editing. All authors contributed to the article and approved the submitted version.

## Conflict of Interest

The authors declare that the research was conducted in the absence of any commercial or financial relationships that could be construed as a potential conflict of interest.

## Publisher’s Note

All claims expressed in this article are solely those of the authors and do not necessarily represent those of their affiliated organizations, or those of the publisher, the editors and the reviewers. Any product that may be evaluated in this article, or claim that may be made by its manufacturer, is not guaranteed or endorsed by the publisher.

## Footnotes

1
https://github.com/rambaut/figtree/releases

2
https://itol.embl.de/
