# Supplementary material for: An Evolutionary Perspective of the Lipocalin Protein Family
Source: Front Physiol. 2021 Aug 23;12:718983. doi: 10.3389/fphys.2021.718983 (PMC8420045; doi:10.3389/fphys.2021.718983)
Supplement: Supplementary file 1 [file Data_Sheet_1.PDF]

CLASS/CLADE	ORDER	SPECIES
Ascidiacea	Enterogona	Ciona intestinalis
Chondrichthyes	Chimaeriformes	Callorhinchus milii
Actinopteri	Semionotiformes	Lepisosteus oculatus
	Cypriniformes	Danio rerio
	Salmoniformes	Salmo salar
	Syngnathiformes	Hippocampus comes
	Spariformes	Sparus aurata
	Pleuronectiformes	Scophthalmus maximus
Coelacanthimorpha	Coelacanthiformes	Latimeria chalumnae
Archelosauria	Crocodylia	Crocodylus porosus
Aves	Galliformes	Gallus gallus
	Casuariiformes	Dromaius novaehollandiae
Mammalia	Dasyuromorphia	Sarcophilus harrisii
	Proboscidea	Loxodonta africana
	Perissodactyla	Equus caballus
	Primates	Homo sapiens
	Rodentia	Mus musculus

LIPOCALIN	CLASS/CLADE	ORDER	SPECIES	SYMBOL	ENSEMBL CODE
AMBP	Chondrichthyes	Chimaeriformes	Callorhinchus milii	AMBP_Cmil	ENSCMIG00000015299
AMBP	Archelosauria	Crocodylia	Crocodylus porosus	AMBP_Cpor	ENSCPRG00005011115
AMBP	Aves	Casuariiformes	Dromaius novaehollandiae	AMBP_Dnov	ENSDNVG00000003449
AMBP	Actinopteri	Cypriniformes	Danio rerio	AMBP_Drer	ENSDARG00000004296
AMBP	Mammalia	Perissodactyla	Equus caballus	AMBP_Ecab	ENSECAG00000008365
AMBP	Aves	Galliformes	Gallus gallus	AMBP_Ggal	ENSGALG00000030255
AMBP	Actinopteri	Syngnathiformes	Hippocampus comes	AMBP_Hcom	ENSHCOG00000002676
AMBP	Mammalia	Primates	Homo sapiens	AMBP_Hsap	ENSG00000106927.12
AMBP	Mammalia	Proboscidea	Loxodonta africana	AMBP_Lafr	ENSLAFG00000027008
AMBP	Coelacanthimorpha	Coelacanthiformes	Latimeria chalumnae	AMBP_Lcha	ENSLACG00000003165
AMBP	Actinopteri	Semionotiformes	Lepisosteus oculatus	AMBP_Locu	ENSLOCG00000002312
AMBP	Mammalia	Rodentia	Mus musculus	AMBP_Mmus	ENSMUSG00000028356
AMBP	Mammalia	Dasyuromorphia	Sarcophilus harrisii	AMBP_Shar	ENSSHAG00000015813
AMBP	Actinopteri	Spariformes	Sparus aurata	AMBP-1_Saur	ENSSAUG00010015153
AMBP	Actinopteri	Pleuronectiformes	Scophthalmus maximus	AMBP-1_Smax	ENSSMAG00000019819
AMBP	Actinopteri	Salmoniformes	Salmo salar	AMBP-1a_Ssal	ENSSSAG00000010137
AMBP	Actinopteri	Salmoniformes	Salmo salar	AMBP-1b_Ssal	ENSSSAG00000042557
AMBP	Actinopteri	Spariformes	Sparus aurata	AMBP-2_Saur	ENSSAUG00010015157
AMBP	Actinopteri	Pleuronectiformes	Scophthalmus maximus	AMBP-2_Smax	ENSSMAG00000019833
APOD	Ascidiacea	Enterogona	Ciona intestinalis	APOD_Cint	ENSCING00000010079
APOD	Archelosauria	Crocodylia	Crocodylus porosus	APOD_Cpor	ENSCPRG00005013493
APOD	Aves	Casuariiformes	Dromaius novaehollandiae	APOD_Dnov	ENSDNVG00000016210
APOD	Mammalia	Perissodactyla	Equus caballus	APOD_Ecab	ENSECAG00000022047
APOD	Aves	Galliformes	Gallus gallus	APOD_Ggal	ENSGALG00000006995
APOD	Mammalia	Primates	Homo sapiens	APOD_Hsap	ENSG00000189058
APOD	Mammalia	Proboscidea	Loxodonta africana	APOD_Lafr	ENSLAFG00000012014
APOD	Coelacanthimorpha	Coelacanthiformes	Latimeria chalumnae	APOD_Lcha	ENSLACG00000022488
APOD	Mammalia	Rodentia	Mus musculus	APOD_Mmus	ENSMUSG00000022548.14
APOD	Mammalia	Dasyuromorphia	Sarcophilus harrisii	APOD_Shar	ENSSHAG00000008795
APOD	Actinopteri	Cypriniformes	Danio rerio	APOD-1_Drer	ENSDARG00000060350
APOD	Actinopteri	Semionotiformes	Lepisosteus oculatus	APOD-1_Locu	ENSLOCG00000007087
APOD	Chondrichthyes	Chimaeriformes	Callorhinchus milii	APOD-1a_Cmil	ENSCMIG00000008971
APOD	Actinopteri	Spariformes	Sparus aurata	APOD-1a_Saur	ENSSAUG00010022065
APOD	Actinopteri	Pleuronectiformes	Scophthalmus maximus	APOD-1a_Smax	ENSSMAG00000007526
APOD	Actinopteri	Salmoniformes	Salmo salar	APOD-1a_Ssal	ENSSSAG00000004976
APOD	Chondrichthyes	Chimaeriformes	Callorhinchus milii	APOD-1b_Cmil	ENSCMIG00000008976
APOD	Actinopteri	Spariformes	Sparus aurata	APOD-1b_Saur	ENSSAUG00010027356
APOD	Actinopteri	Pleuronectiformes	Scophthalmus maximus	APOD-1b_Smax	ENSSMAG00000015827
APOD	Actinopteri	Salmoniformes	Salmo salar	APOD-1b_Ssal	ENSSSAG00000062033
APOD	Actinopteri	Spariformes	Sparus aurata	APOD-1c_Saur	ENSSAUG00010027355
APOD	Actinopteri	Pleuronectiformes	Scophthalmus maximus	APOD-1c_Smax	ENSSMAG00000015800
APOD	Actinopteri	Semionotiformes	Lepisosteus oculatus	APOD-2_Locu	ENSLOCG00000007072
APOD	Actinopteri	Cypriniformes	Danio rerio	APOD-2a_Drer	ENSDARG00000057437
APOD	Actinopteri	Syngnathiformes	Hippocampus comes	APOD-2a_Hcom	ENSHCOG00000011798
APOD	Actinopteri	Spariformes	Sparus aurata	APOD-2a_Saur	ENSSAUG00010019399
APOD	Actinopteri	Pleuronectiformes	Scophthalmus maximus	APOD-2a_Smax	ENSSMAG00000006569
APOD	Actinopteri	Salmoniformes	Salmo salar	APOD-2a_Ssal	ENSSSAG00000077912
APOD	Actinopteri	Cypriniformes	Danio rerio	APOD-2b_Drer	ENSDARG00000060345
APOD	Actinopteri	Syngnathiformes	Hippocampus comes	APOD-2b_Hcom	ENSHCOG00000002829
APOD	Actinopteri	Spariformes	Sparus aurata	APOD-2b_Saur	ENSSAUG00010027357
APOD	Actinopteri	Pleuronectiformes	Scophthalmus maximus	APOD-2b_Smax	ENSSMAG00000015837
APOD	Actinopteri	Salmoniformes	Salmo salar	APOD-2b_Ssal	ENSSSAG00000077906
APOD	Actinopteri	Salmoniformes	Salmo salar	APOD-2c_Ssal	ENSSSAG00000004972
APOM	Actinopteri	Cypriniformes	Danio rerio	APOM_Drer	ENSDARG00000076838
APOM	Mammalia	Perissodactyla	Equus caballus	APOM_Ecab	ENSECAG00000015824
APOM	Mammalia	Primates	Homo sapiens	APOM_Hsap	ENSG00000204444.11
APOM	Mammalia	Proboscidea	Loxodonta africana	APOM_Lafr	ENSLAFG00000009494
APOM	Coelacanthimorpha	Coelacanthiformes	Latimeria chalumnae	APOM_Lcha	ENSLACG00000004283
APOM	Mammalia	Rodentia	Mus musculus	APOM_Mmus	ENSMUSG00000024391
APOM	Actinopteri	Spariformes	Sparus aurata	APOM_Saur	ENSSAUG00010013650
APOM	Actinopteri	Pleuronectiformes	Scophthalmus maximus	APOM_Smax	ENSSMAG00000016541
APOM	Actinopteri	Salmoniformes	Salmo salar	APOM_Ssal	ENSSSAG00000053770
C8G	Chondrichthyes	Chimaeriformes	Callorhinchus milii	C8G_Cmil	ENSCMIG00000015308
C8G	Archelosauria	Crocodylia	Crocodylus porosus	C8G_Cpor	ENSCPRG00005011067
C8G	Aves	Casuariiformes	Dromaius novaehollandiae	C8G_Dnov	ENSDNVG00000003100

C8G	Actinopteri	Cypriniformes	Danio rerio	C8G_Drer	ENSDARG00000032098
C8G	Mammalia	Perissodactyla	Equus caballus	C8G_Ecab	ENSECAG00000021738
C8G	Aves	Galliformes	Gallus gallus	C8G_Ggal	ENSGALG00000040644
C8G	Actinopteri	Syngnathiformes	Hippocampus comes	C8G_Hcom	ENSHCOG00000000341
C8G	Mammalia	Primates	Homo sapiens	C8G_Hsap	ENSG00000176919.13
C8G	Mammalia	Proboscidea	Loxodonta africana	C8G_Lafr	ENSLAFG00000028173
C8G	Coelacanthimorpha	Coelacanthiformes	Latimeria chalumnae	C8G_Lcha	ENSLACG00000022223
C8G	Actinopteri	Semionotiformes	Lepisosteus oculatus	C8G_Locu	ENSLOCG00000003306
C8G	Mammalia	Rodentia	Mus musculus	C8G_Mmus	ENSMUSG00000015083.11
C8G	Actinopteri	Spariformes	Sparus aurata	C8G_Saur	ENSSAUG00010015199
C8G	Mammalia	Dasyuromorphia	Sarcophilus harrisii	C8G_Shar	ENSSHAG00000008097
C8G	Actinopteri	Pleuronectiformes	Scophthalmus maximus	C8G_Smax	ENSSMAG00000018605
C8G	Actinopteri	Salmoniformes	Salmo salar	C8G_Ssal	ENSSSAG00000070566
LCN1	Mammalia	Primates	Homo sapiens	LCN1-1_Hsap	ENSG00000160349
LCN1	Mammalia	Proboscidea	Loxodonta africana	LCN1-1_Lafr	ENSLAFG00000022982
LCN1	Mammalia	Rodentia	Mus musculus	LCN1-1_Mmus	ENSMUSG00000026936
LCN1	Mammalia	Perissodactyla	Equus caballus	LCN1-1a_Ecab	ENSECAG00000032975
LCN1	Mammalia	Dasyuromorphia	Sarcophilus harrisii	LCN1-1a_Shar	ENSSHAG00000006281
LCN1	Mammalia	Perissodactyla	Equus caballus	LCN1-1b_Ecab	ENSECAG00000029752
LCN1	Mammalia	Dasyuromorphia	Sarcophilus harrisii	LCN1-1b_Shar	ENSSHAG00000000734
LCN1	Mammalia	Dasyuromorphia	Sarcophilus harrisii	LCN1-1c_Shar	ENSSHAG00000001933
LCN1	Mammalia	Proboscidea	Loxodonta africana	LCN1-2_Lafr	ENSLAFG00000030625
LCN1	Mammalia	Perissodactyla	Equus caballus	LCN1-2a_Ecab	ENSECAG00000022200
LCN1	Mammalia	Primates	Homo sapiens	LCN1-2a_Hsap	ENSG00000171102.14
LCN1	Mammalia	Rodentia	Mus musculus	LCN1-2a_Mmus	ENSMUSG00000079539
LCN1	Mammalia	Perissodactyla	Equus caballus	LCN1-2b_Ecab	ENSECAG00000008816
LCN1	Mammalia	Primates	Homo sapiens	LCN1-2b_Hsap	ENSG00000122136.13
LCN1	Mammalia	Rodentia	Mus musculus	LCN1-2b_Mmus	ENSMUSG00000062061
LCN1	Mammalia	Rodentia	Mus musculus	LCN1-3_Mmus	ENSMUSG00000026919
LCN2	Mammalia	Perissodactyla	Equus caballus	LCN2_Ecab	ENSECAG00000020957
LCN2	Mammalia	Primates	Homo sapiens	LCN2_Hsap	ENSG00000148346.12
LCN2	Mammalia	Proboscidea	Loxodonta africana	LCN2_Lafr	ENSLAFG00000023195
LCN2	Mammalia	Rodentia	Mus musculus	LCN2_Mmus	ENSMUSG00000026822.14
LCN2	Mammalia	Dasyuromorphia	Sarcophilus harrisii	LCN2_Shar	ENSSHAG00000008440
LCN8	Mammalia	Perissodactyla	Equus caballus	LCN8_Ecab	ENSECAG00000015825
LCN8	Mammalia	Primates	Homo sapiens	LCN8_Hsap	ENSG00000204001
LCN8	Mammalia	Dasyuromorphia	Sarcophilus harrisii	LCN8_Shar	ENSSHAG00000001189
LCN8	Mammalia	Proboscidea	Loxodonta africana	LCN8-1_Lafr	ENSLAFG00000031608
LCN8	Mammalia	Rodentia	Mus musculus	LCN8-1_Mmus	ENSMUSG00000026937.12
LCN8	Mammalia	Proboscidea	Loxodonta africana	LCN8-2_Lafr	ENSLAFG00000028177
LCN8	Mammalia	Rodentia	Mus musculus	LCN8-2_Mmus	ENSMUSG00000036449
LCN9	Mammalia	Primates	Homo sapiens	LCN9_Hsap	ENSG00000148386
LCN9	Mammalia	Perissodactyla	Equus caballus	LCN9-1_Ecab	ENSECAG00000000866
LCN9	Mammalia	Proboscidea	Loxodonta africana	LCN9-1_Lafr	ENSLAFG00000028938
LCN9	Mammalia	Rodentia	Mus musculus	LCN9-1_Mmus	ENSMUSG00000069080
LCN9	Mammalia	Dasyuromorphia	Sarcophilus harrisii	LCN9-1a_Shar	ENSSHAG00000004569
LCN9	Mammalia	Dasyuromorphia	Sarcophilus harrisii	LCN9-1b_Shar	ENSSHAG00000001410
LCN9	Mammalia	Dasyuromorphia	Sarcophilus harrisii	LCN9-1c_Shar	ENSSHAG00000003729
LCN9	Mammalia	Perissodactyla	Equus caballus	LCN9-2_Ecab	ENSECAG00000005458
LCN9	Mammalia	Proboscidea	Loxodonta africana	LCN9-2_Lafr	ENSLAFG00000030623
LCN9	Mammalia	Rodentia	Mus musculus	LCN9-2_Mmus	ENSMUSG00000023210
LCN9	Mammalia	Dasyuromorphia	Sarcophilus harrisii	LCN9-2a_Shar	ENSSHAG00000004149
LCN9	Mammalia	Dasyuromorphia	Sarcophilus harrisii	LCN9-2b_Shar	ENSSHAG00000008301
LCN9	Mammalia	Rodentia	Mus musculus	LCN9-3_Mmus	ENSMUSG00000041333
LCN9	Mammalia	Perissodactyla	Equus caballus	LCN9-3a_Ecab	ENSECAG00000034517
LCN9	Mammalia	Rodentia	Mus musculus	LCN9-3a_Mmus	ENSMUSG00000078689
LCN9	Mammalia	Perissodactyla	Equus caballus	LCN9-3b_Ecab	ENSECAG00000035661
LCN9	Mammalia	Rodentia	Mus musculus	LCN9-3b_Mmus	ENSMUSG00000058523
LCN9	Mammalia	Rodentia	Mus musculus	LCN9-3c_Mmus	ENSMUSG00000066154.11
LCN9	Mammalia	Rodentia	Mus musculus	LCN9-3d_Mmus	ENSMUSG00000066153
LCN9	Mammalia	Rodentia	Mus musculus	LCN9-3e_Mmus	ENSMUSG00000078672
LCN9	Mammalia	Rodentia	Mus musculus	LCN9-3f_Mmus	ENSMUSG00000096688
LCN9	Mammalia	Rodentia	Mus musculus	LCN9-3g_Mmus	ENSMUSG00000078673.10
LCN9	Mammalia	Rodentia	Mus musculus	LCN9-3h_Mmus	ENSMUSG00000107188
LCN9	Mammalia	Rodentia	Mus musculus	LCN9-3i_Mmus	ENSMUSG00000078686.11

LCN9	Mammalia	Rodentia	Mus musculus	LCN9-3j_Mmus	ENSMUSG00000078683
LCN9	Mammalia	Rodentia	Mus musculus	LCN9-3k_Mmus	ENSMUSG00000073842.10
LCN9	Mammalia	Rodentia	Mus musculus	LCN9-3l_Mmus	ENSMUSG00000078687
LCN9	Mammalia	Rodentia	Mus musculus	LCN9-3m_Mmus	ENSMUSG00000106922
LCN9	Mammalia	Rodentia	Mus musculus	LCN9-3n_Mmus	ENSMUSG00000089873
LCN9	Mammalia	Rodentia	Mus musculus	LCN9-3o_Mmus	ENSMUSG00000106700
LCN9	Mammalia	Rodentia	Mus musculus	LCN9-3p_Mmus	ENSMUSG00000073830.11
LCN9	Mammalia	Rodentia	Mus musculus	LCN9-3q_Mmus	ENSMUSG00000078674
LCN9	Mammalia	Rodentia	Mus musculus	LCN9-3r_Mmus	ENSMUSG00000078675
LCN9	Mammalia	Rodentia	Mus musculus	LCN9-3s_Mmus	ENSMUSG00000106882
LCN9	Mammalia	Rodentia	Mus musculus	LCN9-3t_Mmus	ENSMUSG00000073834.10
LCN9	Mammalia	Rodentia	Mus musculus	LCN9-3u_Mmus	ENSMUSG00000110439
LCN9	Mammalia	Rodentia	Mus musculus	LCN9-3v_Mmus	ENSMUSG00000078680
LCN9	Mammalia	Rodentia	Mus musculus	LCN9-3w_Mmus	ENSMUSG00000078688.11
LCN9	Mammalia	Rodentia	Mus musculus	LCN9-3x_Mmus	ENSMUSG00000094793
LCN9	Mammalia	Rodentia	Mus musculus	LCN9-3y_Mmus	ENSMUSG00000107311
LCN9	Mammalia	Rodentia	Mus musculus	LCN9-3z_Mmus	ENSMUSG00000096674
LCN10	Mammalia	Perissodactyla	Equus caballus	LCN10_Ecab	ENSECAG00000013784
LCN10	Mammalia	Primates	Homo sapiens	LCN10_Hsap	ENSG00000187922.14
LCN10	Mammalia	Rodentia	Mus musculus	LCN10_Mmus	ENSMUSG00000047356
LCN10	Mammalia	Proboscidea	Loxodonta africana	LCN10-1a_Lafr	ENSLAFG00000005661
LCN10	Mammalia	Proboscidea	Loxodonta africana	LCN10-1b_Lafr	ENSLAFG00000030186
LCN12	Mammalia	Perissodactyla	Equus caballus	LCN12_Ecab	ENSECAG00000022475
LCN12	Mammalia	Primates	Homo sapiens	LCN12_Hsap	ENSG00000184925.12
LCN12	Mammalia	Proboscidea	Loxodonta africana	LCN12_Lafr	ENSLAFG00000005099
LCN12	Mammalia	Rodentia	Mus musculus	LCN12_Mmus	ENSMUSG00000026943.12
LCN15	Mammalia	Primates	Homo sapiens	LCN15_Hsap	ENSG00000177984
LCN15	Mammalia	Dasyuromorphia	Sarcophilus harrisii	LCN15_Shar	ENSSHAG00000002167
LCN15	Aves	Casuariiformes	Dromaius novaehollandiae	LCN15-1_Dnov	ENSDNVG00000003067
LCN15	Mammalia	Perissodactyla	Equus caballus	LCN15-1_Ecab	ENSECAG00000042990
LCN15	Aves	Galliformes	Gallus gallus	LCN15-1_Ggal	ENSGALG00000043064
LCN15	Mammalia	Proboscidea	Loxodonta africana	LCN15-1_Lafr	ENSLAFG00000030069
LCN15	Aves	Casuariiformes	Dromaius novaehollandiae	LCN15-2_Dnov	ENSDNVG00000003080
LCN15	Mammalia	Perissodactyla	Equus caballus	LCN15-2_Ecab	ENSECAG00000039055
LCN15	Aves	Galliformes	Gallus gallus	LCN15-2_Ggal	ENSGALG00000032170
LCN15	Mammalia	Proboscidea	Loxodonta africana	LCN15-2_Lafr	ENSLAFG00000014812
ORM	Aves	Casuariiformes	Dromaius novaehollandiae	ORM_Dnov	ENSDNVG00000019103
ORM	Aves	Galliformes	Gallus gallus	ORM_Ggal	ENSGALG00000005099
ORM	Mammalia	Dasyuromorphia	Sarcophilus harrisii	ORM_Shar	ENSSHAG00000003219
ORM	Mammalia	Perissodactyla	Equus caballus	ORM-1_Ecab	ENSECAG00000036967
ORM	Mammalia	Primates	Homo sapiens	ORM1_Hsap	ENSG00000229314
ORM	Mammalia	Proboscidea	Loxodonta africana	ORM-1_Lafr	ENSLAFG00000000508
ORM	Mammalia	Rodentia	Mus musculus	ORM-1a_Mmus	ENSMUSG00000039196
ORM	Mammalia	Rodentia	Mus musculus	ORM-1b_Mmus	ENSMUSG00000061540
ORM	Mammalia	Rodentia	Mus musculus	ORM-1c_Mmus	ENSMUSG00000028359.4
ORM	Mammalia	Perissodactyla	Equus caballus	ORM-2_Ecab	ENSECAG00000036100
ORM	Mammalia	Primates	Homo sapiens	ORM2_Hsap	ENSG00000228278
ORM	Mammalia	Proboscidea	Loxodonta africana	ORM-2_Lafr	ENSLAFG00000013705
ORM	Mammalia	Perissodactyla	Equus caballus	ORM-3_Ecab	ENSECAG00000036760
ORM	Mammalia	Perissodactyla	Equus caballus	ORM-4_Ecab	ENSECAG00000035262
PAEP	Mammalia	Primates	Homo sapiens	PAEP_Hsap	ENSG00000122133.17
PAEP	Mammalia	Proboscidea	Loxodonta africana	PAEP_Lafr	ENSLAFG00000028561
PAEP	Mammalia	Dasyuromorphia	Sarcophilus harrisii	PAEP_Shar	ENSSHAG00000006105
PAEP	Mammalia	Perissodactyla	Equus caballus	PAEP-1a_Ecab	ENSECAG00000012852
PAEP	Mammalia	Perissodactyla	Equus caballus	PAEP-1b_Ecab	ENSECAG00000009820
PTGDS	Chondrichthyes	Chimaeriformes	Callorhinchus milii	PTGDS_Cmil	ENSCMIG00000015302
PTGDS	Archelosauria	Crocodylia	Crocodylus porosus	PTGDS_Cpor	ENSCPRG00005011064
PTGDS	Aves	Casuariiformes	Dromaius novaehollandiae	PTGDS_Dnov	ENSDNVG00000003088
PTGDS	Mammalia	Perissodactyla	Equus caballus	PTGDS_Ecab	ENSECAG00000009262
PTGDS	Aves	Galliformes	Gallus gallus	PTGDS_Ggal	ENSGALG00000030886
PTGDS	Mammalia	Primates	Homo sapiens	PTGDS_Hsap	ENSG00000107317.13
PTGDS	Mammalia	Proboscidea	Loxodonta africana	PTGDS_Lafr	ENSLAFG00000003721
PTGDS	Coelacanthimorpha	Coelacanthiformes	Latimeria chalumnae	PTGDS_Lcha	ENSLACG00000002612

PTGDS	Actinopteri	Semionotiformes	Lepisosteus oculatus	PTGDS_Locu	ENSLOCG00000002295
PTGDS	Mammalia	Rodentia	Mus musculus	PTGDS_Mmus	ENSMUSG00000015090.13
PTGDS	Mammalia	Dasyuromorphia	Sarcophilus harrisii	PTGDS_Shar	ENSSHAG00000008790
PTGDS	Actinopteri	Syngnathiformes	Hippocampus comes	PTGDS-1_Hcom	ENSHCOG00000010942
PTGDS	Actinopteri	Spariformes	Sparus aurata	PTGDS-1_Saur	ENSSAUG00010015198
PTGDS	Actinopteri	Pleuronectiformes	Scophthalmus maximus	PTGDS-1_Smax	ENSSMAG00000018620
PTGDS	Actinopteri	Salmoniformes	Salmo salar	PTGDS-1_Ssal	ENSSSAG00000079841
PTGDS	Actinopteri	Cypriniformes	Danio rerio	PTGDS-1a_Drer	ENSDARG00000027088
PTGDS	Actinopteri	Cypriniformes	Danio rerio	PTGDS-1b_Drer	ENSDARG00000071626
PTGDS	Actinopteri	Cypriniformes	Danio rerio	PTGDS-2_Drer	ENSDARG00000045979
PTGDS	Actinopteri	Syngnathiformes	Hippocampus comes	PTGDS-2_Hcom	ENSHCOG00000020922
PTGDS	Actinopteri	Spariformes	Sparus aurata	PTGDS-2_Saur	ENSSAUG00010001857
PTGDS	Actinopteri	Pleuronectiformes	Scophthalmus maximus	PTGDS-2_Smax	ENSSMAG00000012487
PTGDS	Actinopteri	Salmoniformes	Salmo salar	PTGDS-2a_Ssal	ENSSSAG00000004300
PTGDS	Actinopteri	Salmoniformes	Salmo salar	PTGDS-2b_Ssal	ENSSSAG00000000779
PTGDS	Actinopteri	Salmoniformes	Salmo salar	PTGDS-2c_Ssal	ENSSSAG00000000767
PTGDS	Actinopteri	Syngnathiformes	Hippocampus comes	PTGDS-3_Hcom	ENSHCOG00000002711
PTGDS	Actinopteri	Spariformes	Sparus aurata	PTGDS-3_Saur	ENSSAUG00010015164
PTGDS	Actinopteri	Pleuronectiformes	Scophthalmus maximus	PTGDS-3_Smax	ENSSMAG00000019825
PTGDS	Actinopteri	Cypriniformes	Danio rerio	PTGDS-3a_Drer	ENSDARG00000069439
PTGDS	Actinopteri	Salmoniformes	Salmo salar	PTGDS-3a_Ssal	ENSSSAG00000010165
PTGDS	Actinopteri	Cypriniformes	Danio rerio	PTGDS-3b_Drer	ENSDARG00000067851
PTGDS	Actinopteri	Salmoniformes	Salmo salar	PTGDS-3b_Ssal	ENSSSAG00000067643
RBP4	Chondrichthyes	Chimaeriformes	Callorhinchus milii	RBP4_Cmil	ENSCMIG00000013498
RBP4	Mammalia	Perissodactyla	Equus caballus	RBP4_Ecab	ENSECAG00000034419
RBP4	Mammalia	Primates	Homo sapiens	RBP4_Hsap	ENSG00000138207.14
RBP4	Mammalia	Proboscidea	Loxodonta africana	RBP4_Lafr	ENSLAFG00000015168
RBP4	Mammalia	Rodentia	Mus musculus	RBP4_Mmus	ENSMUSG00000024990.13
RBP4	Archelosauria	Crocodylia	Crocodylus porosus	RBP4-1_Cpor	ENSCPRG00005017094
RBP4	Aves	Casuariiformes	Dromaius novaehollandiae	RBP4-1_Dnov	ENSDNVG00000004244
RBP4	Actinopteri	Cypriniformes	Danio rerio	RBP4-1_Drer	ENSDARG00000101199
RBP4	Aves	Galliformes	Gallus gallus	RBP4-1_Ggal	ENSGALG00000006629
RBP4	Actinopteri	Syngnathiformes	Hippocampus comes	RBP4-1_Hcom	ENSHCOG00000002918
RBP4	Coelacanthimorpha	Coelacanthiformes	Latimeria chalumnae	RBP4-1_Lcha	ENSLACG00000016645
RBP4	Actinopteri	Semionotiformes	Lepisosteus oculatus	RBP4-1_Locu	ENSLOCG00000005811
RBP4	Actinopteri	Spariformes	Sparus aurata	RBP4-1_Saur	ENSSAUG00010017699
RBP4	Mammalia	Dasyuromorphia	Sarcophilus harrisii	RBP4-1_Shar	ENSSHAG00000016992
RBP4	Actinopteri	Pleuronectiformes	Scophthalmus maximus	RBP4-1_Smax	ENSSMAG00000019074
RBP4	Actinopteri	Salmoniformes	Salmo salar	RBP4-1a_Ssal	ENSSSAG00000007721
RBP4	Actinopteri	Salmoniformes	Salmo salar	RBP4-1b_Ssal	ENSSSAG00000062385
RBP4	Archelosauria	Crocodylia	Crocodylus porosus	RBP4-2_Cpor	ENSCPRG00005014298
RBP4	Aves	Casuariiformes	Dromaius novaehollandiae	RBP4-2_Dnov	ENSDNVG00000000943
RBP4	Actinopteri	Cypriniformes	Danio rerio	RBP4-2_Drer	ENSDARG00000044684
RBP4	Aves	Galliformes	Gallus gallus	RBP4-2_Ggal	ENSGALG00000015374
RBP4	Coelacanthimorpha	Coelacanthiformes	Latimeria chalumnae	RBP4-2_Lcha	ENSLACG00000000895
RBP4	Actinopteri	Semionotiformes	Lepisosteus oculatus	RBP4-2_Locu	ENSLOCG00000012234
RBP4	Mammalia	Dasyuromorphia	Sarcophilus harrisii	RBP4-2_Shar	ENSSHAG00000014584
RBP4	Actinopteri	Syngnathiformes	Hippocampus comes	RBP4-2a_Hcom	ENSHCOG00000003205
RBP4	Actinopteri	Spariformes	Sparus aurata	RBP4-2a_Saur	ENSSAUG00010022404
RBP4	Actinopteri	Pleuronectiformes	Scophthalmus maximus	RBP4-2a_Smax	ENSSMAG00000020171
RBP4	Actinopteri	Salmoniformes	Salmo salar	RBP4-2a_Ssal	ENSSSAG00000075142
RBP4	Actinopteri	Syngnathiformes	Hippocampus comes	RBP4-2b_Hcom	ENSHCOG00000011618
RBP4	Actinopteri	Spariformes	Sparus aurata	RBP4-2b_Saur	ENSSAUG00010005607
RBP4	Actinopteri	Pleuronectiformes	Scophthalmus maximus	RBP4-2b_Smax	ENSSMAG00000019120
RBP4	Actinopteri	Salmoniformes	Salmo salar	RBP4-2b_Ssal	ENSSSAG00000081425
RBP4	Actinopteri	Salmoniformes	Salmo salar	RBP4-2c_Ssal	ENSSSAG00000062846
