# Supplementary material for: An Evolutionary Perspective of the Lipocalin Protein Family
Source: Front Physiol. 2021 Aug 23;12:718983. doi: 10.3389/fphys.2021.718983 (PMC8420045; doi:10.3389/fphys.2021.718983)
Supplement: Supplementary file 2 [file Data_Sheet_2.PDF]

(LCN_Cint:0.51320325,((((((((RBP4-2_Cpor:0.150248,RBP4-2_Shar:0.149239):0.033333,RBP4-
2_Lcha:0.176349):0.061283,RBP4-2_Dnov:0.007752):0.014925,RBP4-2_Ggal:3.0E-6):3.0E-6,(((RBP4-
2_Drer:0.045592,((((RBP4-2a_Smax:0.029512,RBP4-2a_Hcom:0.05261):0.008152,RBP4-
2a_Saur:0.019266):0.018602,((RBP4-2c_Ssal:0.014525,RBP4-2b_Ssal:0.012127):0.05731,(RBP4-
2b_Saur:0.054726,(RBP4-2b_Hcom:0.025314,RBP4-
2b_Smax:0.076381):0.037992):0.039223):0.028462):0.008598,RBP4-
2a_Ssal:0.027988):0.019765):0.044429,RBP4-
2_Locu:0.047988):0.061554,RBP4_Cmil:0.163721):0.073169):0.333836,(((RBP4-1_Cpor:0.157278,(RBP4-
1_Dnov:0.020249,RBP4-1_Ggal:0.004384):0.052259):0.045526,
(((RBP4_Ecab:0.033535,RBP4_Lafr:0.107666):0.027514,RBP4_Hsap:2.0E-6):0.018057,
(RBP4_Mmus:0.120661,RBP4-1_Shar:0.206984):0.028274):0.074265):0.102305,((((RBP4-1_Drer:0.114639,RBP4-
1_Locu:0.139292):0.041455,(RBP4-1a_Ssal:0.02422,RBP4-1b_Ssal:0.013273):0.103391):0.019898,(RBP4-
1_Saur:0.153649,(RBP4-1_Hcom:0.213412,RBP4-1_Smax:0.222328):0.043366):0.03543):0.259501,RBP4-
1_Lcha:0.344601):0.049335):0.296628):0.795978,((((((((PTGDS_Cpor:0.143561,
(PTGDS_Dnov:0.038846,PTGDS_Ggal:0.186873):0.175):0.161859,((PTGDS_Shar:0.363103,
(((PTGDS_Ecab:0.23638,PTGDS_Lafr:0.141413):0.025409,PTGDS_Hsap:0.089067):0.058263,PTGDS_Mmus:0.1
87983):0.31597):0.210905,(LCN2_Shar:0.392919,((((LCN2_Ecab:0.11696,(LCN12_Lafr:0.765883,
((LCN12_Ecab:0.373082,LCN12_Mmus:0.438418):0.022499,LCN12_Hsap:0.216918):0.856994):0.28246):0.04814
8,LCN2_Mmus:0.284257):0.048738,LCN2_Hsap:0.269439):0.047391,LCN2_Lafr:0.122515):0.222566):0.799677):
0.119211):0.221082,(((((PTGDS-1a_Drer:0.044516,PTGDS-1b_Drer:0.211392):0.17852,(((PTGDS-
1_Smax:0.185973,PTGDS-1_Saur:0.137428):0.029925,PTGDS-1_Hcom:0.334818):0.045251,PTGDS-
1_Ssal:0.221519):0.068253):0.105121,(PTGDS-2_Drer:0.288054,(((PTGDS-2_Smax:0.144369,PTGDS-
2_Saur:0.088309):0.039889,PTGDS-2_Hcom:0.214567):0.058169,((PTGDS-2b_Ssal:0.059502,PTGDS-
2c_Ssal:0.546967):0.059864,PTGDS-
2a_Ssal:0.066604):0.070512):0.047553):0.064365):0.477225,PTGDS_Cmil:0.524985):0.104774,(((((PTGDS-
3b_Drer:0.524532,PTGDS-3b_Ssal:0.727627):0.155396,(((PTGDS-3_Smax:0.123235,PTGDS-
3_Saur:0.198265):0.051177,PTGDS-3_Hcom:0.079788):0.158552,PTGDS-
3a_Ssal:0.114445):0.049183):0.089891,PTGDS_Locu:0.183006):0.049471,PTGDS-3a_Drer:0.336327):0.6471,
(((((AMBP_Cpor:0.309309,(AMBP_Dnov:0.056744,AMBP_Ggal:0.091769):0.167821):0.165222,
(AMBP_Shar:0.321845,((AMBP_Ecab:0.147713,
(AMBP_Hsap:0.070318,AMBP_Mmus:0.21779):0.058284):0.052555,AMBP_Lafr:0.072402):0.182482):0.180193):
0.18245,AMBP_Lcha:0.264201):0.116062,(AMBP_Locu:0.333812,(AMBP_Drer:0.184587,((((AMBP-
1_Smax:0.131197,AMBP-1_Saur:0.13728):0.031419,AMBP_Hcom:0.360667):0.069274,(AMBP-
2_Saur:0.148136,AMBP-2_Smax:0.415156):0.531973):0.091788,(AMBP-1a_Ssal:0.076658,AMBP-
1b_Ssal:0.075771):0.129281):0.132426):0.414418):0.022795):0.194125,AMBP_Cmil:0.992079):0.292763):0.08470
6):0.126301):0.066297,PTGDS_Lcha:0.623012):0.145791,(((((LCN15-2_Dnov:0.313202,LCN15-
2_Ggal:0.310405):0.372508,(LCN15_Shar:0.299712,((LCN15-
2_Ecab:0.119177,LCN15_Hsap:0.120085):0.026199,LCN15-2_Lafr:0.090893):0.073932):0.505906):0.05776,
(LCN15-1_Ecab:0.392344,LCN15-1_Lafr:0.05553):0.869246):0.194284,(LCN15-1_Dnov:0.304711,LCN15-
1_Ggal:0.479681):0.689853):0.094685,(((LCN8-1_Lafr:0.502387,LCN8-1_Mmus:0.730362):0.526076,
(LCN8_Shar:0.563796,(LCN8_Ecab:0.104918,(LCN8-2_Lafr:0.148147,(LCN8_Hsap:0.174081,LCN8-
2_Mmus:0.312035):0.138489):0.055852):0.757032):0.179243):0.537762,((LCN10_Ecab:0.193788,
(LCN10_Hsap:0.221709,LCN10_Mmus:0.153734):0.121355):0.053462,(LCN10-1a_Lafr:0.09505,LCN10-
1b_Lafr:0.211061):0.124968):1.22473):0.346963):0.196915):0.059899,((C8G_Cpor:0.195691,
((C8G_Ggal:0.331174,C8G_Dnov:0.228372):0.15453,(C8G_Shar:0.266642,((C8G_Ecab:0.09011,
(C8G_Hsap:0.077301,C8G_Mmus:0.219442):0.052568):0.017746,C8G_Lafr:0.105809):0.344909):0.225338):0.076
665):0.110796,((C8G_Lcha:0.28187,C8G_Cmil:0.695048):0.065464,(C8G_Locu:0.316918,
((((C8G_Smax:0.083067,C8G_Saur:0.147608):0.048909,C8G_Hcom:0.133678):0.187054,C8G_Ssal:0.176754):0.4
46982,C8G_Drer:0.530349):0.261528):0.184894):0.056493):1.181318):0.093838,(((((LCN1-
1b_Shar:0.373228,LCN1-1c_Shar:0.901264):0.274717,LCN1-1a_Shar:1.14243):0.030378,(((LCN1-
1b_Ecab:0.02731,LCN1-1a_Ecab:2.0E-6):0.268987,LCN1-1_Hsap:0.338872):0.174044,LCN1-
1_Lafr:0.303461):0.217215):0.088311,((((LCN1-2b_Ecab:0.050544,LCN1-2a_Ecab:0.040547):0.229869,(LCN1-
2_Lafr:0.339502,(LCN1-2b_Hsap:0.145301,LCN1-2a_Hsap:0.046287):0.718016):0.060558):0.082504,LCN1-
3_Mmus:1.110378):0.07764,(LCN1-2b_Mmus:0.558197,LCN1-
2a_Mmus:0.255359):0.240955):0.08575):0.172054,LCN1-1_Mmus:0.700091):0.958836):0.091141,((PAEP-
like_Shar:1.133004,((LCN9-1_Mmus:0.985234,(((LCN9-1b_Shar:0.287701,LCN9-
1c_Shar:0.686112):0.219981,LCN9-1a_Shar:0.45362):0.415339,(((((LCN9-2_Ecab:0.250219,(LCN9-

1_Lafr:0.362507,LCN9_Hsap:0.237548):0.055798):0.074673,LCN9-2_Mmus:0.372579):0.441087,(((LCN9-
3a_Ecab:0.115178,LCN9-3b_Ecab:0.168445):0.286468,LCN9-2_Lafr:0.671505):0.05793,(LCN9-
3_Mmus:0.043047,((LCN9-3b_Mmus:0.092622,((LCN9-3e_Mmus:0.060375,((((LCN9-3p_Mmus:3.0E-6,LCN9-
3o_Mmus:3.0E-6):0.006247,((((LCN9-3t_Mmus:0.0,LCN9-3s_Mmus:0.0):0.0,LCN9-3r_Mmus:0.0):2.0E-6,LCN9-
3q_Mmus:3.0E-6):2.0E-6,((LCN9-3w_Mmus:3.0E-6,(LCN9-3x_Mmus:0.006245,(LCN9-3z_Mmus:3.0E-6,LCN9-
3y_Mmus:3.0E-6):2.0E-6):0.006244):0.006241,(LCN9-3v_Mmus:2.0E-6,LCN9-3u_Mmus:3.0E-6):0.00625):3.0E-
6):3.0E-6):2.0E-6,(LCN9-3g_Mmus:0.026687,(((LCN9-3k_Mmus:0.005018,((LCN9-3n_Mmus:3.0E-6,LCN9-
3m_Mmus:3.0E-6):0.015104,LCN9-3l_Mmus:0.010078):2.0E-6):0.005029,LCN9-3j_Mmus:0.005027):2.0E-6,
(LCN9-3i_Mmus:3.0E-6,LCN9-3h_Mmus:3.0E-6):2.0E-6):0.031791):0.019595):0.012036,LCN9-
3f_Mmus:0.006554):0.145394):0.02263,(LCN9-3d_Mmus:0.035449,LCN9-
3c_Mmus:0.036704):0.211215):0.009569):0.030326,LCN9-
3a_Mmus:0.08924):0.029891):0.296857):0.286612):0.187643,(LCN9-2a_Shar:0.693214,LCN9-
2b_Shar:0.935583):0.196634):0.088183,LCN9-1_Ecab:0.760559):0.191336):0.138441):0.11019,
(PAEP_Shar:1.3202,(((PAEP-1b_Ecab:0.106099,PAEP_Hsap:0.636514):0.192664,PAEP-
1a_Ecab:0.158868):0.214281,PAEP_Lafr:0.322085):0.261939):0.756776):0.344287):0.076275,
(((ORM_Dnov:0.256498,ORM_Ggal:0.30313):0.64896,(ORM_Shar:0.718452,((((ORM-4_Ecab:0.34671,ORM-
1_Ecab:0.150165):0.038881,(ORM-2_Ecab:0.115758,ORM-3_Ecab:0.165561):0.09299):0.158927,
((ORM2_Hsap:0.048842,ORM1_Hsap:0.067465):0.236015,(ORM-1_Lafr:0.110322,ORM-
2_Lafr:0.109451):0.17695):0.094551):0.124203,((ORM-1c_Mmus:0.163364,ORM-
1b_Mmus:0.06312):0.05715,ORM-1a_Mmus:0.074315):0.448011):0.263406):0.543624):0.56239,
((APOM_Lcha:0.47746,
(((APOM_Ecab:0.056211,APOM_Hsap:0.045102):0.015846,APOM_Lafr:0.068793):0.017925,APOM_Mmus:0.12
2297):1.037987):0.110609,
(((APOM_Smax:0.188119,APOM_Saur:0.289708):0.331463,APOM_Ssal:0.436628):0.170986,APOM_Drer:1.4723
81):0.305812):0.96948):1.02442):0.117616):0.983591):1.234494,(APOD_Cpor:0.294842,(APOD_Ggal:0.105995,
(APOD_Dnov:0.034114,(((APOD_Shar:0.198959,
((APOD_Ecab:0.073139,APOD_Lafr:0.052384):0.019358,APOD_Mmus:0.182598):0.042872):0.09146,APOD_Hsa
p:0.079401):0.147604,(((((APOD-2a_Drer:0.222807,(((APOD-2a_Smax:0.192374,APOD-
2a_Saur:0.103293):0.078304,APOD-2a_Hcom:0.137499):0.109983,(APOD-2b_Ssal:0.013246,APOD-
2a_Ssal:2.0E-6):0.116332):0.148825):0.099222,(APOD-2b_Drer:0.10608,(((APOD-2b_Smax:0.077271,APOD-
2b_Hcom:0.169614):0.038883,APOD-2b_Saur:0.06826):0.062222,APOD-
2c_Ssal:0.089304):0.049309):0.265555):0.154003,APOD-2_Locu:0.199129):0.14535,((APOD-1_Locu:0.352854,
((APOD-1a_Smax:0.143193,APOD-1a_Saur:0.1108):0.344245,(APOD-1_Drer:0.421201,(((APOD-
1c_Smax:0.034157,APOD-1c_Saur:0.109181):0.206461,(APOD-1b_Smax:0.049069,APOD-
1b_Saur:0.180488):0.141717):0.087731,(APOD-1a_Ssal:0.176393,APOD-
1b_Ssal:0.529151):0.074044):0.18282):0.123438):0.080279):0.075163,(APOD-1a_Cmil:0.29824,APOD-
1b_Cmil:0.7906):0.825852):0.086973):0.083087,APOD_Lcha:0.542326):0.135993):0.008278125):0.008278125):0.
01655625):0.0331125):1.0264065);

```
#NEXUS
begin taxa;
	dimensions ntax=19;
	taxlabels
	AMBP-1_Saur
	AMBP-1_Smax
	AMBP-1a_Ssal
	AMBP-1b_Ssal
	AMBP-2_Saur
	AMBP-2_Smax
	AMBP_Cmil
	AMBP_Cpor
	AMBP_Dnov
	AMBP_Drer
	AMBP_Ecab
	AMBP_Ggal
	AMBP_Hcom
	AMBP_Hsap
	AMBP_Lafr
	AMBP_Lcha
	AMBP_Locu
	AMBP_Mmus
	AMBP_Shar
;
end;

begin trees;
	tree tree_1 = [&R] (AMBP_Drer:0.20227,((((AMBP-1_Smax:0.136527,AMBP-1_Saur:0.137962)
[&Bootstrap=72]:0.03277,AMBP_Hcom:0.368881)[&Bootstrap=92,!rotate=false]:0.086428,(AMBP-
2_Saur:0.141065,AMBP-2_Smax:0.462975)[&Bootstrap=100,!rotate=true]:0.484785)
[&Bootstrap=89,!rotate=true]:0.09254,(AMBP-1a_Ssal:0.077357,AMBP-1b_Ssal:0.077648)
[&Bootstrap=99]:0.131065)[&Bootstrap=95]:0.153328,(AMBP_Locu:0.348805,((AMBP_Lcha:0.247355,
(((AMBP_Dnov:0.070857,AMBP_Ggal:0.078438)[&Bootstrap=98]:0.145192,AMBP_Cpor:0.379263)
[&Bootstrap=98]:0.188097,(AMBP_Shar:0.352042,((AMBP_Ecab:0.155401,
(AMBP_Hsap:0.068312,AMBP_Mmus:0.227005)[&Bootstrap=96,!rotate=true]:0.058465)
[&Bootstrap=96]:0.059213,AMBP_Lafr:0.070272)[&Bootstrap=100]:0.192381)[&Bootstrap=97]:0.188923)
[&Bootstrap=99]:0.2392)[&Bootstrap=83,!rotate=false]:0.13147,AMBP_Cmil:1.291186)
[&Bootstrap=70,!rotate=false]:0.042257)[&Bootstrap=100,!rotate=false]:0.438537);
end;

begin figtree;
	set appearance.backgroundColorAttribute="Default";
	set appearance.backgroundColour=#ffffff;
	set appearance.branchColorAttribute="User selection";
	set appearance.branchColorGradient=false;
	set appearance.branchLineWidth=1.0;
	set appearance.branchMinLineWidth=0.0;
	set appearance.branchWidthAttribute="Fixed";
	set appearance.foregroundColour=#000000;
	set appearance.hilightingGradient=false;
	set appearance.selectionColour=#2d3680;
	set branchLabels.colorAttribute="User selection";
	set branchLabels.displayAttribute="Branch times";
	set branchLabels.fontName="Adobe Devanagari";
	set branchLabels.fontSize=8;
	set branchLabels.fontStyle=0;
	set branchLabels.isShown=false;
```

```
set branchLabels.significantDigits=4;
set layout.expansion=0;
set layout.layoutType="RECTILINEAR";
set layout.zoom=0;
set legend.attribute="Bootstrap";
set legend.fontSize=10.0;
set legend.isShown=false;
set legend.significantDigits=4;
set nodeBars.barWidth=4.0;
set nodeBars.displayAttribute=null;
set nodeBars.isShown=false;
set nodeLabels.colorAttribute="User selection";
set nodeLabels.displayAttribute="Bootstrap";
set nodeLabels.fontName="Trebuchet MS";
set nodeLabels.fontSize=14;
set nodeLabels.fontStyle=0;
set nodeLabels.isShown=true;
set nodeLabels.significantDigits=4;
set nodeShapeExternal.colourAttribute="User selection";
set nodeShapeExternal.isShown=false;
set nodeShapeExternal.minSize=10.0;
set nodeShapeExternal.scaleType=Width;
set nodeShapeExternal.shapeType=Circle;
set nodeShapeExternal.size=4.0;
set nodeShapeExternal.sizeAttribute="Fixed";
set nodeShapeInternal.colourAttribute="User selection";
set nodeShapeInternal.isShown=false;
set nodeShapeInternal.minSize=10.0;
set nodeShapeInternal.scaleType=Width;
set nodeShapeInternal.shapeType=Circle;
set nodeShapeInternal.size=4.0;
set nodeShapeInternal.sizeAttribute="Fixed";
set polarLayout.alignTipLabels=false;
set polarLayout.angularRange=0;
set polarLayout.rootAngle=0;
set polarLayout.rootLength=100;
set polarLayout.showRoot=true;
set radialLayout.spread=0.0;
set rectilinearLayout.alignTipLabels=false;
set rectilinearLayout.curvature=0;
set rectilinearLayout.rootLength=100;
set scale.offsetAge=0.0;
set scale.rootAge=1.0;
set scale.scaleFactor=1.0;
set scale.scaleRoot=false;
set scaleAxis.automaticScale=true;
set scaleAxis.fontSize=8.0;
set scaleAxis.isShown=false;
set scaleAxis.lineWidth=1.0;
set scaleAxis.majorTicks=1.0;
set scaleAxis.minorTicks=0.5;
set scaleAxis.origin=0.0;
set scaleAxis.reverseAxis=false;
set scaleAxis.showGrid=true;
set scaleBar.automaticScale=true;
set scaleBar.fontSize=10.0;
set scaleBar.isShown=true;
```

```
	set scaleBar.lineWidth=1.0;
	set scaleBar.scaleRange=0.0;
	set tipLabels.colorAttribute="User selection";
	set tipLabels.displayAttribute="Names";
	set tipLabels.fontName="Trebuchet MS";
	set tipLabels.fontSize=14;
	set tipLabels.fontStyle=0;
	set tipLabels.isShown=true;
	set tipLabels.significantDigits=4;
	set trees.order=true;
	set trees.orderType="increasing";
	set trees.rooting=true;
	set trees.rootingType="User Selection";
	set trees.transform=false;
	set trees.transformType="cladogram";
end;
```

```
#NEXUS
begin taxa;
	dimensions ntax=33;
	taxlabels
	APOD-1_Drer
	APOD-1_Locu
	APOD-1a_Cmil
	APOD-1a_Saur
	APOD-1a_Smax
	APOD-1a_Ssal
	APOD-1b_Cmil
	APOD-1b_Saur
	APOD-1b_Smax
	APOD-1b_Ssal
	APOD-1c_Saur
	APOD-1c_Smax
	APOD-2_Locu
	APOD-2a_Drer
	APOD-2a_Hcom
	APOD-2a_Saur
	APOD-2a_Smax
	APOD-2a_Ssal
	APOD-2b_Drer
	APOD-2b_Hcom
	APOD-2b_Saur
	APOD-2b_Smax
	APOD-2b_Ssal
	APOD-2c_Ssal
	APOD_Cpor
	APOD_Dnov
	APOD_Ecab
	APOD_Ggal
	APOD_Hsap
	APOD_Lafr
	APOD_Lcha
	APOD_Mmus
	APOD_Shar
;
end;

begin trees;
	tree tree_1 = [&R] (APOD-2a_Drer:0.226429,(((APOD-2a_Smax:0.197005,APOD-2a_Saur:0.099358)
[&Bootstrap=77]:0.069119,APOD-2a_Hcom:0.143954)[&Bootstrap=96]:0.122297,(APOD-
2b_Ssal:0.012448,APOD-2a_Ssal:2.0E-6)[&Bootstrap=100]:0.115311)[&Bootstrap=95]:0.123363,((APOD-
2b_Drer:0.10076,(((APOD-2b_Smax:0.066988,APOD-2b_Hcom:0.179806)[&Bootstrap=97]:0.039197,APOD-
2b_Saur:0.065563)[&Bootstrap=99]:0.069775,APOD-2c_Ssal:0.082783)[&Bootstrap=97]:0.051348)
[&Bootstrap=100]:0.287879,(APOD-2_Locu:0.212908,((((APOD_Cpor:0.302439,
(APOD_Dnov:0.194093,APOD_Ggal:0.108825)[&Bootstrap=97]:0.108006)[&Bootstrap=97]:0.162501,
((APOD_Shar:0.18937,((APOD_Ecab:0.070178,APOD_Lafr:0.050776)
[&Bootstrap=79]:0.018382,APOD_Mmus:0.186774)[&Bootstrap=88]:0.047113)
[&Bootstrap=96]:0.08356,APOD_Hsap:0.079597)[&Bootstrap=96]:0.15144)
[&Bootstrap=96]:0.15307,APOD_Lcha:0.584084)[&Bootstrap=50]:0.066986,((APOD-1_Locu:0.397695,((APOD-
1a_Smax:0.145489,APOD-1a_Saur:0.111276)[&Bootstrap=100]:0.336835,(APOD-1_Drer:0.450343,(((APOD-
1c_Smax:0.031381,APOD-1c_Saur:0.104815)[&Bootstrap=100,!rotate=true]:0.203482,(APOD-
1b_Smax:0.044721,APOD-1b_Saur:0.184041)[&Bootstrap=100,!rotate=true]:0.153213)[&Bootstrap=55]:0.07593,
(APOD-1a_Ssal:0.190717,APOD-1b_Ssal:0.562596)[&Bootstrap=91,!rotate=true]:0.066296)
[&Bootstrap=99]:0.173737)[&Bootstrap=96]:0.144251)[&Bootstrap=87]:0.082156)[&Bootstrap=84]:0.0813,
```

```
(APOD-1a_Cmil[&!rotate=false]:0.301439,APOD-1b_Cmil[&!rotate=true]:1.005605)
[&Bootstrap=100,!rotate=false]:0.996998)[&Bootstrap=56,!rotate=true]:0.088853)[&Bootstrap=98]:0.142074)
[&Bootstrap=99]:0.15512)[&Bootstrap=84]:0.099618);
end;

begin figtree;
	set appearance.backgroundColorAttribute="Default";
	set appearance.backgroundColour=#ffffff;
	set appearance.branchColorAttribute="User selection";
	set appearance.branchColorGradient=false;
	set appearance.branchLineWidth=1.0;
	set appearance.branchMinLineWidth=0.0;
	set appearance.branchWidthAttribute="Fixed";
	set appearance.foregroundColour=#000000;
	set appearance.hilightingGradient=false;
	set appearance.selectionColour=#2d3680;
	set branchLabels.colorAttribute="User selection";
	set branchLabels.displayAttribute="Branch times";
	set branchLabels.fontName="Adobe Devanagari";
	set branchLabels.fontSize=8;
	set branchLabels.fontStyle=0;
	set branchLabels.isShown=false;
	set branchLabels.significantDigits=4;
	set layout.expansion=0;
	set layout.layoutType="RECTILINEAR";
	set layout.zoom=0;
	set legend.attribute="Bootstrap";
	set legend.fontSize=10.0;
	set legend.isShown=false;
	set legend.significantDigits=4;
	set nodeBars.barWidth=4.0;
	set nodeBars.displayAttribute=null;
	set nodeBars.isShown=false;
	set nodeLabels.colorAttribute="User selection";
	set nodeLabels.displayAttribute="Bootstrap";
	set nodeLabels.fontName="Trebuchet MS";
	set nodeLabels.fontSize=14;
	set nodeLabels.fontStyle=0;
	set nodeLabels.isShown=true;
	set nodeLabels.significantDigits=4;
	set nodeShapeExternal.colourAttribute="User selection";
	set nodeShapeExternal.isShown=false;
	set nodeShapeExternal.minSize=10.0;
	set nodeShapeExternal.scaleType=Width;
	set nodeShapeExternal.shapeType=Circle;
	set nodeShapeExternal.size=4.0;
	set nodeShapeExternal.sizeAttribute="Fixed";
	set nodeShapeInternal.colourAttribute="User selection";
	set nodeShapeInternal.isShown=false;
	set nodeShapeInternal.minSize=10.0;
	set nodeShapeInternal.scaleType=Width;
	set nodeShapeInternal.shapeType=Circle;
	set nodeShapeInternal.size=4.0;
	set nodeShapeInternal.sizeAttribute="Fixed";
	set polarLayout.alignTipLabels=false;
	set polarLayout.angularRange=0;
	set polarLayout.rootAngle=0;
```

```
	set polarLayout.rootLength=100;
	set polarLayout.showRoot=true;
	set radialLayout.spread=0.0;
	set rectilinearLayout.alignTipLabels=false;
	set rectilinearLayout.curvature=0;
	set rectilinearLayout.rootLength=100;
	set scale.offsetAge=0.0;
	set scale.rootAge=1.0;
	set scale.scaleFactor=1.0;
	set scale.scaleRoot=false;
	set scaleAxis.automaticScale=true;
	set scaleAxis.fontSize=8.0;
	set scaleAxis.isShown=false;
	set scaleAxis.lineWidth=1.0;
	set scaleAxis.majorTicks=1.0;
	set scaleAxis.minorTicks=0.5;
	set scaleAxis.origin=0.0;
	set scaleAxis.reverseAxis=false;
	set scaleAxis.showGrid=true;
	set scaleBar.automaticScale=true;
	set scaleBar.fontSize=10.0;
	set scaleBar.isShown=true;
	set scaleBar.lineWidth=1.0;
	set scaleBar.scaleRange=0.0;
	set tipLabels.colorAttribute="User selection";
	set tipLabels.displayAttribute="Names";
	set tipLabels.fontName="Trebuchet MS";
	set tipLabels.fontSize=14;
	set tipLabels.fontStyle=0;
	set tipLabels.isShown=true;
	set tipLabels.significantDigits=4;
	set trees.order=true;
	set trees.orderType="increasing";
	set trees.rooting=true;
	set trees.rootingType="User Selection";
	set trees.transform=false;
	set trees.transformType="cladogram";
end;
```

```
#NEXUS
begin taxa;
	dimensions ntax=9;
	taxlabels
	APOM_Drer
	APOM_Ecab
	APOM_Hsap
	APOM_Lafr
	APOM_Lcha
	APOM_Mmus
	APOM_Saur
	APOM_Smax
	APOM_Ssal
;
end;

begin trees;
	tree tree_1 = [&R] (APOM_Drer[&!rotate=true]:1.279684,((APOM_Smax:0.18987,APOM_Saur:0.258894)
[&Bootstrap=96,!rotate=true]:0.267036,APOM_Ssal:0.45172)[&Bootstrap=57]:0.057653,(APOM_Lcha:0.489877,
(((APOM_Ecab:0.052942,APOM_Hsap:0.043459)
[&Bootstrap=84,!rotate=true]:0.014532,APOM_Mmus:0.129791)
[&Bootstrap=72]:0.025059,APOM_Lafr:0.041266)[&Bootstrap=100]:0.792255)
[&Bootstrap=97,!rotate=true]:0.374021);
end;

begin figtree;
	set appearance.backgroundColorAttribute="Default";
	set appearance.backgroundColour=#ffffff;
	set appearance.branchColorAttribute="User selection";
	set appearance.branchColorGradient=false;
	set appearance.branchLineWidth=1.0;
	set appearance.branchMinLineWidth=0.0;
	set appearance.branchWidthAttribute="Fixed";
	set appearance.foregroundColour=#000000;
	set appearance.hilightingGradient=false;
	set appearance.selectionColour=#2d3680;
	set branchLabels.colorAttribute="User selection";
	set branchLabels.displayAttribute="Branch times";
	set branchLabels.fontName="Adobe Devanagari";
	set branchLabels.fontSize=8;
	set branchLabels.fontStyle=0;
	set branchLabels.isShown=false;
	set branchLabels.significantDigits=4;
	set layout.expansion=0;
	set layout.layoutType="RECTILINEAR";
	set layout.zoom=0;
	set legend.attribute="Bootstrap";
	set legend.fontSize=10.0;
	set legend.isShown=false;
	set legend.significantDigits=4;
	set nodeBars.barWidth=4.0;
	set nodeBars.displayAttribute=null;
	set nodeBars.isShown=false;
	set nodeLabels.colorAttribute="User selection";
	set nodeLabels.displayAttribute="Bootstrap";
	set nodeLabels.fontName="Trebuchet MS";
	set nodeLabels.fontSize=14;
```

```
set nodeLabels.fontStyle=0;
set nodeLabels.isShown=true;
set nodeLabels.significantDigits=4;
set nodeShapeExternal.colourAttribute="User selection";
set nodeShapeExternal.isShown=false;
set nodeShapeExternal.minSize=10.0;
set nodeShapeExternal.scaleType=Width;
set nodeShapeExternal.shapeType=Circle;
set nodeShapeExternal.size=4.0;
set nodeShapeExternal.sizeAttribute="Fixed";
set nodeShapeInternal.colourAttribute="User selection";
set nodeShapeInternal.isShown=false;
set nodeShapeInternal.minSize=10.0;
set nodeShapeInternal.scaleType=Width;
set nodeShapeInternal.shapeType=Circle;
set nodeShapeInternal.size=4.0;
set nodeShapeInternal.sizeAttribute="Fixed";
set polarLayout.alignTipLabels=false;
set polarLayout.angularRange=0;
set polarLayout.rootAngle=0;
set polarLayout.rootLength=100;
set polarLayout.showRoot=true;
set radialLayout.spread=0.0;
set rectilinearLayout.alignTipLabels=false;
set rectilinearLayout.curvature=0;
set rectilinearLayout.rootLength=100;
set scale.offsetAge=0.0;
set scale.rootAge=1.0;
set scale.scaleFactor=1.0;
set scale.scaleRoot=false;
set scaleAxis.automaticScale=true;
set scaleAxis.fontSize=8.0;
set scaleAxis.isShown=false;
set scaleAxis.lineWidth=1.0;
set scaleAxis.majorTicks=1.0;
set scaleAxis.minorTicks=0.5;
set scaleAxis.origin=0.0;
set scaleAxis.reverseAxis=false;
set scaleAxis.showGrid=true;
set scaleBar.automaticScale=true;
set scaleBar.fontSize=10.0;
set scaleBar.isShown=true;
set scaleBar.lineWidth=1.0;
set scaleBar.scaleRange=0.0;
set tipLabels.colorAttribute="User selection";
set tipLabels.displayAttribute="Names";
set tipLabels.fontName="Trebuchet MS";
set tipLabels.fontSize=14;
set tipLabels.fontStyle=0;
set tipLabels.isShown=true;
set tipLabels.significantDigits=4;
set trees.order=false;
set trees.orderType="increasing";
set trees.rooting=true;
set trees.rootingType="User Selection";
set trees.transform=false;
set trees.transformType="cladogram";
```

end;

```
#NEXUS
begin taxa;
	dimensions ntax=16;
	taxlabels
	C8G_Cmil
	C8G_Cpor
	C8G_Dnov
	C8G_Drer
	C8G_Ecab
	C8G_Ggal
	C8G_Hcom
	C8G_Hsap
	C8G_Lafr
	C8G_Lcha
	C8G_Locu
	C8G_Mmus
	C8G_Saur
	C8G_Shar
	C8G_Smax
	C8G_Ssal
;
end;

begin trees;
	tree tree_1 = [&R] (C8G_Drer:0.580343,(((C8G_Smax:0.078508,C8G_Saur:0.1564)
[&Bootstrap=93,!rotate=true]:0.049429,C8G_Hcom:0.140313)[&Bootstrap=99]:0.18883,C8G_Ssal:0.176013)
[&Bootstrap=100]:0.487509,(C8G_Locu:0.295955,(((C8G_Cpor:0.186373,
((C8G_Ggal:0.353218,C8G_Dnov:0.235583)[&Bootstrap=88]:0.134747,(C8G_Shar:0.267108,
((C8G_Ecab:0.093059,(C8G_Hsap:0.077613,C8G_Mmus:0.223495)[&Bootstrap=95,!rotate=true]:0.050448)
[&Bootstrap=83]:0.014767,C8G_Lafr:0.109449)[&Bootstrap=100]:0.391512)[&Bootstrap=100]:0.256491)
[&Bootstrap=69]:0.0912)[&Bootstrap=92]:0.182849,C8G_Lcha:0.353857)
[&Bootstrap=64]:0.064693,C8G_Cmil:0.732892)[&Bootstrap=93]:0.177869)[&Bootstrap=89]:0.288518);
end;

begin figtree;
	set appearance.backgroundColorAttribute="Default";
	set appearance.backgroundColour=#ffffff;
	set appearance.branchColorAttribute="User selection";
	set appearance.branchColorGradient=false;
	set appearance.branchLineWidth=1.0;
	set appearance.branchMinLineWidth=0.0;
	set appearance.branchWidthAttribute="Fixed";
	set appearance.foregroundColour=#000000;
	set appearance.hilightingGradient=false;
	set appearance.selectionColour=#2d3680;
	set branchLabels.colorAttribute="User selection";
	set branchLabels.displayAttribute="Branch times";
	set branchLabels.fontName="Adobe Devanagari";
	set branchLabels.fontSize=8;
	set branchLabels.fontStyle=0;
	set branchLabels.isShown=false;
	set branchLabels.significantDigits=4;
	set layout.expansion=0;
	set layout.layoutType="RECTILINEAR";
	set layout.zoom=0;
	set legend.attribute=null;
	set legend.fontSize=10.0;
```

```
set legend.isShown=false;
set legend.significantDigits=4;
set nodeBars.barWidth=4.0;
set nodeBars.displayAttribute=null;
set nodeBars.isShown=false;
set nodeLabels.colorAttribute="User selection";
set nodeLabels.displayAttribute="Bootstrap";
set nodeLabels.fontName="Trebuchet MS";
set nodeLabels.fontSize=14;
set nodeLabels.fontStyle=0;
set nodeLabels.isShown=true;
set nodeLabels.significantDigits=4;
set nodeShapeExternal.colourAttribute=null;
set nodeShapeExternal.isShown=false;
set nodeShapeExternal.minSize=10.0;
set nodeShapeExternal.scaleType=Width;
set nodeShapeExternal.shapeType=Circle;
set nodeShapeExternal.size=4.0;
set nodeShapeExternal.sizeAttribute=null;
set nodeShapeInternal.colourAttribute=null;
set nodeShapeInternal.isShown=false;
set nodeShapeInternal.minSize=10.0;
set nodeShapeInternal.scaleType=Width;
set nodeShapeInternal.shapeType=Circle;
set nodeShapeInternal.size=4.0;
set nodeShapeInternal.sizeAttribute=null;
set polarLayout.alignTipLabels=false;
set polarLayout.angularRange=0;
set polarLayout.rootAngle=0;
set polarLayout.rootLength=100;
set polarLayout.showRoot=true;
set radialLayout.spread=0.0;
set rectilinearLayout.alignTipLabels=false;
set rectilinearLayout.curvature=0;
set rectilinearLayout.rootLength=100;
set scale.offsetAge=0.0;
set scale.rootAge=1.0;
set scale.scaleFactor=1.0;
set scale.scaleRoot=false;
set scaleAxis.automaticScale=true;
set scaleAxis.fontSize=8.0;
set scaleAxis.isShown=false;
set scaleAxis.lineWidth=1.0;
set scaleAxis.majorTicks=1.0;
set scaleAxis.minorTicks=0.5;
set scaleAxis.origin=0.0;
set scaleAxis.reverseAxis=false;
set scaleAxis.showGrid=true;
set scaleBar.automaticScale=true;
set scaleBar.fontSize=10.0;
set scaleBar.isShown=true;
set scaleBar.lineWidth=1.0;
set scaleBar.scaleRange=0.0;
set tipLabels.colorAttribute="User selection";
set tipLabels.displayAttribute="Names";
set tipLabels.fontName="Trebuchet MS";
set tipLabels.fontSize=14;
```

```
	set tipLabels.fontStyle=0;
	set tipLabels.isShown=true;
	set tipLabels.significantDigits=4;
	set trees.order=true;
	set trees.orderType="increasing";
	set trees.rooting=true;
	set trees.rootingType="User Selection";
	set trees.transform=false;
	set trees.transformType="cladogram";
end;
```

```
#NEXUS
begin taxa;
	dimensions ntax=16;
	taxlabels
	LCN1-1_Hsap
	LCN1-1_Lafr
	LCN1-1_Mmus
	LCN1-1a_Ecab
	LCN1-1a_Shar
	LCN1-1b_Ecab
	LCN1-1b_Shar
	LCN1-1c_Shar
	LCN1-2_Lafr
	LCN1-2a_Ecab
	LCN1-2a_Hsap
	LCN1-2a_Mmus
	LCN1-2b_Ecab
	LCN1-2b_Hsap
	LCN1-2b_Mmus
	LCN1-3_Mmus
;
end;

begin trees;
	tree tree_1 = [&R] (LCN1-1b_Shar[&!rotate=true]:0.431118,((((((LCN1-2b_Ecab:0.049763,LCN1-
2a_Ecab:0.039607)[&Bootstrap=100]:0.22486,(LCN1-2_Lafr:0.331973,(LCN1-2b_Hsap:0.169963,LCN1-
2a_Hsap:0.018946)[&Bootstrap=100]:0.762027)[&Bootstrap=75]:0.065873)[&Bootstrap=62]:0.055646,LCN1-
3_Mmus:1.141851)[&Bootstrap=53]:0.090298,(LCN1-2b_Mmus:0.539859,LCN1-2a_Mmus:0.257777)
[&Bootstrap=96]:0.252222)[&Bootstrap=76]:0.129946,((((LCN1-1b_Ecab:0.026074,LCN1-1a_Ecab:2.0E-6)
[&Bootstrap=100]:0.262516,LCN1-1_Hsap:0.335784)[&Bootstrap=94]:0.165845,LCN1-1_Lafr:0.314075)
[&Bootstrap=83]:0.146436,LCN1-1_Mmus:0.865691)[&Bootstrap=64]:0.070649)
[&Bootstrap=65,!rotate=false]:0.068602,LCN1-1a_Shar:1.204389)[&Bootstrap=95,!rotate=true]:0.245687,LCN1-
1c_Shar:0.896964);
end;

begin figtree;
	set appearance.backgroundColorAttribute="Default";
	set appearance.backgroundColour=#ffffff;
	set appearance.branchColorAttribute="User selection";
	set appearance.branchColorGradient=false;
	set appearance.branchLineWidth=1.0;
	set appearance.branchMinLineWidth=0.0;
	set appearance.branchWidthAttribute="Fixed";
	set appearance.foregroundColour=#000000;
	set appearance.hilightingGradient=false;
	set appearance.selectionColour=#2d3680;
	set branchLabels.colorAttribute="User selection";
	set branchLabels.displayAttribute="Branch times";
	set branchLabels.fontName="Trebuchet MS";
	set branchLabels.fontSize=14;
	set branchLabels.fontStyle=0;
	set branchLabels.isShown=false;
	set branchLabels.significantDigits=4;
	set layout.expansion=0;
	set layout.layoutType="RECTILINEAR";
	set layout.zoom=0;
	set legend.attribute=null;
```

```
set legend.fontSize=10.0;
set legend.isShown=false;
set legend.significantDigits=4;
set nodeBars.barWidth=4.0;
set nodeBars.displayAttribute=null;
set nodeBars.isShown=false;
set nodeLabels.colorAttribute="User selection";
set nodeLabels.displayAttribute="Bootstrap";
set nodeLabels.fontName="Trebuchet MS";
set nodeLabels.fontSize=14;
set nodeLabels.fontStyle=0;
set nodeLabels.isShown=true;
set nodeLabels.significantDigits=4;
set nodeShapeExternal.colourAttribute=null;
set nodeShapeExternal.isShown=false;
set nodeShapeExternal.minSize=10.0;
set nodeShapeExternal.scaleType=Width;
set nodeShapeExternal.shapeType=Circle;
set nodeShapeExternal.size=4.0;
set nodeShapeExternal.sizeAttribute=null;
set nodeShapeInternal.colourAttribute=null;
set nodeShapeInternal.isShown=false;
set nodeShapeInternal.minSize=10.0;
set nodeShapeInternal.scaleType=Width;
set nodeShapeInternal.shapeType=Circle;
set nodeShapeInternal.size=4.0;
set nodeShapeInternal.sizeAttribute=null;
set polarLayout.alignTipLabels=false;
set polarLayout.angularRange=0;
set polarLayout.rootAngle=0;
set polarLayout.rootLength=100;
set polarLayout.showRoot=true;
set radialLayout.spread=0.0;
set rectilinearLayout.alignTipLabels=false;
set rectilinearLayout.curvature=0;
set rectilinearLayout.rootLength=100;
set scale.offsetAge=0.0;
set scale.rootAge=1.0;
set scale.scaleFactor=1.0;
set scale.scaleRoot=false;
set scaleAxis.automaticScale=true;
set scaleAxis.fontSize=8.0;
set scaleAxis.isShown=false;
set scaleAxis.lineWidth=1.0;
set scaleAxis.majorTicks=1.0;
set scaleAxis.minorTicks=0.5;
set scaleAxis.origin=0.0;
set scaleAxis.reverseAxis=false;
set scaleAxis.showGrid=true;
set scaleBar.automaticScale=true;
set scaleBar.fontSize=14.0;
set scaleBar.isShown=true;
set scaleBar.lineWidth=1.0;
set scaleBar.scaleRange=0.0;
set tipLabels.colorAttribute="User selection";
set tipLabels.displayAttribute="Names";
set tipLabels.fontName="Trebuchet MS";
```

```
    set tipLabels.fontSize=14;
    set tipLabels.fontStyle=0;
    set tipLabels.isShown=true;
    set tipLabels.significantDigits=4;
    set trees.order=true;
    set trees.orderType="increasing";
    set trees.rooting=true;
    set trees.rootingType="User Selection";
    set trees.transform=false;
    set trees.transformType="cladogram";
end;
```

```
#NEXUS
begin taxa;
	dimensions ntax=7;
	taxlabels
	LCN8-1_Lafr
	LCN8-1_Mmus
	LCN8-2_Lafr
	LCN8-2_Mmus
	LCN8_Ecab
	LCN8_Hsap
	LCN8_Shar
;
end;

begin trees;
	tree tree_1 = [&R] (LCN8-1_Lafr:0.480177,LCN8-1_Mmus:0.877475,(LCN8_Shar[&!rotate=true]:0.552692,
(LCN8_Ecab:0.105797,(LCN8-2_Lafr:0.154644,(LCN8_Hsap:0.189165,LCN8-2_Mmus:0.315632)
[&Bootstrap=67,!rotate=true]:0.128308)[&Bootstrap=51]:0.057537)[&Bootstrap=100]:0.872494)
[&Bootstrap=100,!rotate=true]:0.829932);
end;

begin figtree;
	set appearance.backgroundColorAttribute="Default";
	set appearance.backgroundColour=#ffffff;
	set appearance.branchColorAttribute="User selection";
	set appearance.branchColorGradient=false;
	set appearance.branchLineWidth=1.0;
	set appearance.branchMinLineWidth=0.0;
	set appearance.branchWidthAttribute="Fixed";
	set appearance.foregroundColour=#000000;
	set appearance.hilightingGradient=false;
	set appearance.selectionColour=#2d3680;
	set branchLabels.colorAttribute="User selection";
	set branchLabels.displayAttribute="Branch times";
	set branchLabels.fontName="Trebuchet MS";
	set branchLabels.fontSize=14;
	set branchLabels.fontStyle=0;
	set branchLabels.isShown=false;
	set branchLabels.significantDigits=4;
	set layout.expansion=0;
	set layout.layoutType="RECTILINEAR";
	set layout.zoom=0;
	set legend.attribute=null;
	set legend.fontSize=10.0;
	set legend.isShown=false;
	set legend.significantDigits=4;
	set nodeBars.barWidth=4.0;
	set nodeBars.displayAttribute=null;
	set nodeBars.isShown=false;
	set nodeLabels.colorAttribute="User selection";
	set nodeLabels.displayAttribute="Bootstrap";
	set nodeLabels.fontName="Trebuchet MS";
	set nodeLabels.fontSize=14;
	set nodeLabels.fontStyle=0;
	set nodeLabels.isShown=true;
	set nodeLabels.significantDigits=4;
	set nodeShapeExternal.colourAttribute=null;
```

```
	set nodeShapeExternal.isShown=false;
	set nodeShapeExternal.minSize=10.0;
	set nodeShapeExternal.scaleType=Width;
	set nodeShapeExternal.shapeType=Circle;
	set nodeShapeExternal.size=4.0;
	set nodeShapeExternal.sizeAttribute=null;
	set nodeShapeInternal.colourAttribute=null;
	set nodeShapeInternal.isShown=false;
	set nodeShapeInternal.minSize=10.0;
	set nodeShapeInternal.scaleType=Width;
	set nodeShapeInternal.shapeType=Circle;
	set nodeShapeInternal.size=4.0;
	set nodeShapeInternal.sizeAttribute=null;
	set polarLayout.alignTipLabels=false;
	set polarLayout.angularRange=0;
	set polarLayout.rootAngle=0;
	set polarLayout.rootLength=100;
	set polarLayout.showRoot=true;
	set radialLayout.spread=0.0;
	set rectilinearLayout.alignTipLabels=false;
	set rectilinearLayout.curvature=0;
	set rectilinearLayout.rootLength=100;
	set scale.offsetAge=0.0;
	set scale.rootAge=1.0;
	set scale.scaleFactor=1.0;
	set scale.scaleRoot=false;
	set scaleAxis.automaticScale=true;
	set scaleAxis.fontSize=8.0;
	set scaleAxis.isShown=false;
	set scaleAxis.lineWidth=1.0;
	set scaleAxis.majorTicks=1.0;
	set scaleAxis.minorTicks=0.5;
	set scaleAxis.origin=0.0;
	set scaleAxis.reverseAxis=false;
	set scaleAxis.showGrid=true;
	set scaleBar.automaticScale=true;
	set scaleBar.fontSize=14.0;
	set scaleBar.isShown=true;
	set scaleBar.lineWidth=1.0;
	set scaleBar.scaleRange=0.0;
	set tipLabels.colorAttribute="User selection";
	set tipLabels.displayAttribute="Names";
	set tipLabels.fontName="Trebuchet MS";
	set tipLabels.fontSize=14;
	set tipLabels.fontStyle=0;
	set tipLabels.isShown=true;
	set tipLabels.significantDigits=4;
	set trees.order=true;
	set trees.orderType="increasing";
	set trees.rooting=true;
	set trees.rootingType="User Selection";
	set trees.transform=false;
	set trees.transformType="cladogram";
end;
```

```
#NEXUS
begin taxa;
	dimensions ntax=41;
	taxlabels
	LCN9-1_Ecab
	LCN9-1_Lafr
	LCN9-1_Mmus
	LCN9-1a_Shar
	LCN9-1b_Shar
	LCN9-1c_Shar
	LCN9-2_Ecab
	LCN9-2_Lafr
	LCN9-2_Mmus
	LCN9-2a_Shar
	LCN9-2b_Shar
	LCN9-3_Mmus
	LCN9-3a_Ecab
	LCN9-3a_Mmus
	LCN9-3b_Ecab
	LCN9-3b_Mmus
	LCN9-3c_Mmus
	LCN9-3d_Mmus
	LCN9-3e_Mmus
	LCN9-3f_Mmus
	LCN9-3g_Mmus
	LCN9-3h_Mmus
	LCN9-3i_Mmus
	LCN9-3j_Mmus
	LCN9-3k_Mmus
	LCN9-3l_Mmus
	LCN9-3m_Mmus
	LCN9-3n_Mmus
	LCN9-3o_Mmus
	LCN9-3p_Mmus
	LCN9-3q_Mmus
	LCN9-3r_Mmus
	LCN9-3s_Mmus
	LCN9-3t_Mmus
	LCN9-3u_Mmus
	LCN9-3v_Mmus
	LCN9-3w_Mmus
	LCN9-3x_Mmus
	LCN9-3y_Mmus
	LCN9-3z_Mmus
	LCN9_Hsap
;
end;

begin trees;
	tree tree_1 = [&R] (LCN9-1b_Shar:0.349588,LCN9-1a_Shar[&!rotate=true]:0.653665,(LCN9-
1c_Shar:0.60122,((((((LCN9-2_Ecab:0.265818,(LCN9-1_Lafr:0.392609,LCN9_Hsap:0.268535)
[&Bootstrap=79]:0.058543)[&Bootstrap=89,!rotate=true]:0.073049,LCN9-2_Mmus:0.372702)
[&Bootstrap=100]:0.456027,(((LCN9-3a_Ecab:0.106811,LCN9-3b_Ecab:0.191514)
[&Bootstrap=100,!rotate=true]:0.307989,LCN9-2_Lafr:0.612744)[&Bootstrap=69]:0.037552,(LCN9-
3_Mmus:0.051717,(((LCN9-3b_Mmus:0.092857,(((LCN9-3p_Mmus:3.0E-6,LCN9-3o_Mmus:3.0E-6)
[&Bootstrap=94]:0.006285,(((((LCN9-3t_Mmus:0.0,LCN9-3r_Mmus:0.0):0.0,LCN9-3s_Mmus:0.0):3.0E-6,
((LCN9-3w_Mmus:3.0E-6,(LCN9-3x_Mmus:0.006258,(LCN9-3z_Mmus:3.0E-6,LCN9-3y_Mmus:3.0E-6)
```

```
[&Bootstrap=73]:3.0E-6)[&Bootstrap=56]:0.00626)[&Bootstrap=61,!rotate=true]:0.00626,(LCN9-
3g_Mmus:0.040921,(((LCN9-3k_Mmus:2.0E-6,(LCN9-3l_Mmus:0.0093,(LCN9-3n_Mmus:2.0E-6,LCN9-
3m_Mmus:2.0E-6)[&Bootstrap=100]:0.013893)[&Bootstrap=63,!rotate=true]:0.004605)
[&Bootstrap=59,!rotate=true]:0.004623,LCN9-3j_Mmus:2.0E-6)[&Bootstrap=50]:0.00462,(LCN9-3i_Mmus:3.0E-
6,LCN9-3h_Mmus:3.0E-6)[&Bootstrap=60]:3.0E-6)[&Bootstrap=100]:0.047404)
[&Bootstrap=79,!rotate=true]:0.019666)[&Bootstrap=12]:3.0E-6)[&Bootstrap=7]:3.0E-6,(LCN9-3v_Mmus:4.0E-
6,LCN9-3u_Mmus:2.0E-6)[&Bootstrap=98]:0.006268)[&Bootstrap=9]:3.0E-6,LCN9-3q_Mmus:2.0E-6)
[&Bootstrap=18]:3.0E-6)[&Bootstrap=95]:0.013022,LCN9-3f_Mmus:0.006393)[&Bootstrap=100]:0.16365)
[&Bootstrap=47,!rotate=true]:0.007246,(LCN9-3e_Mmus:0.057102,(LCN9-3d_Mmus:0.038877,LCN9-
3c_Mmus:0.033357)[&Bootstrap=100]:0.203163)[&Bootstrap=59,!rotate=true]:0.038017)
[&Bootstrap=64]:0.031925,LCN9-3a_Mmus:0.081537)[&Bootstrap=71]:0.035977)
[&Bootstrap=100,!rotate=true]:0.361362)[&Bootstrap=98,!rotate=true]:0.263856)
[&Bootstrap=91,!rotate=true]:0.212994,(LCN9-1_Ecab:0.922201,LCN9-2a_Shar:0.875973)
[&Bootstrap=64]:0.046944)[&Bootstrap=83]:0.143961,LCN9-2b_Shar:0.931604)
[&Bootstrap=65]:0.043267,LCN9-1_Mmus:1.351605)[&Bootstrap=93]:0.323836)
[&Bootstrap=65,!rotate=true]:0.206655);
end;

begin figtree;
	set appearance.backgroundColorAttribute="Default";
	set appearance.backgroundColour=#ffffff;
	set appearance.branchColorAttribute="User selection";
	set appearance.branchColorGradient=false;
	set appearance.branchLineWidth=1.0;
	set appearance.branchMinLineWidth=0.0;
	set appearance.branchWidthAttribute="Fixed";
	set appearance.foregroundColour=#000000;
	set appearance.hilightingGradient=false;
	set appearance.selectionColour=#2d3680;
	set branchLabels.colorAttribute="User selection";
	set branchLabels.displayAttribute="Branch times";
	set branchLabels.fontName="Trebuchet MS";
	set branchLabels.fontSize=14;
	set branchLabels.fontStyle=0;
	set branchLabels.isShown=false;
	set branchLabels.significantDigits=4;
	set layout.expansion=0;
	set layout.layoutType="RECTILINEAR";
	set layout.zoom=1000;
	set legend.attribute=null;
	set legend.fontSize=10.0;
	set legend.isShown=false;
	set legend.significantDigits=4;
	set nodeBars.barWidth=4.0;
	set nodeBars.displayAttribute=null;
	set nodeBars.isShown=false;
	set nodeLabels.colorAttribute="User selection";
	set nodeLabels.displayAttribute="Bootstrap";
	set nodeLabels.fontName="Trebuchet MS";
	set nodeLabels.fontSize=14;
	set nodeLabels.fontStyle=0;
	set nodeLabels.isShown=true;
	set nodeLabels.significantDigits=4;
	set nodeShapeExternal.colourAttribute=null;
	set nodeShapeExternal.isShown=false;
	set nodeShapeExternal.minSize=10.0;
	set nodeShapeExternal.scaleType=Width;
```

```
	set nodeShapeExternal.shapeType=Circle;
	set nodeShapeExternal.size=4.0;
	set nodeShapeExternal.sizeAttribute=null;
	set nodeShapeInternal.colourAttribute=null;
	set nodeShapeInternal.isShown=false;
	set nodeShapeInternal.minSize=10.0;
	set nodeShapeInternal.scaleType=Width;
	set nodeShapeInternal.shapeType=Circle;
	set nodeShapeInternal.size=4.0;
	set nodeShapeInternal.sizeAttribute=null;
	set polarLayout.alignTipLabels=false;
	set polarLayout.angularRange=0;
	set polarLayout.rootAngle=0;
	set polarLayout.rootLength=100;
	set polarLayout.showRoot=true;
	set radialLayout.spread=0.0;
	set rectilinearLayout.alignTipLabels=false;
	set rectilinearLayout.curvature=0;
	set rectilinearLayout.rootLength=100;
	set scale.offsetAge=0.0;
	set scale.rootAge=1.0;
	set scale.scaleFactor=1.0;
	set scale.scaleRoot=false;
	set scaleAxis.automaticScale=true;
	set scaleAxis.fontSize=8.0;
	set scaleAxis.isShown=false;
	set scaleAxis.lineWidth=1.0;
	set scaleAxis.majorTicks=1.0;
	set scaleAxis.minorTicks=0.5;
	set scaleAxis.origin=0.0;
	set scaleAxis.reverseAxis=false;
	set scaleAxis.showGrid=true;
	set scaleBar.automaticScale=true;
	set scaleBar.fontSize=14.0;
	set scaleBar.isShown=true;
	set scaleBar.lineWidth=1.0;
	set scaleBar.scaleRange=0.0;
	set tipLabels.colorAttribute="User selection";
	set tipLabels.displayAttribute="Names";
	set tipLabels.fontName="Trebuchet MS";
	set tipLabels.fontSize=14;
	set tipLabels.fontStyle=0;
	set tipLabels.isShown=true;
	set tipLabels.significantDigits=4;
	set trees.order=false;
	set trees.orderType="increasing";
	set trees.rooting=true;
	set trees.rootingType="User Selection";
	set trees.transform=false;
	set trees.transformType="cladogram";
end;
```

```
#NEXUS
begin taxa;
	dimensions ntax=5;
	taxlabels
	LCN10-1a_Lafr
	LCN10-1b_Lafr
	LCN10_Ecab
	LCN10_Hsap
	LCN10_Mmus
;
end;

begin trees;
	tree tree_1 = [&R] (LCN10_Ecab:0.19433,(LCN10_Hsap:0.2068,LCN10_Mmus:0.149849)
[&Bootstrap=98]:0.10478,(LCN10-1a_Lafr:0.088291,LCN10-1b_Lafr:0.196394)
[&Bootstrap=100,!rotate=true]:0.168563);
end;

begin figtree;
	set appearance.backgroundColorAttribute="Default";
	set appearance.backgroundColour=#ffffff;
	set appearance.branchColorAttribute="User selection";
	set appearance.branchColorGradient=false;
	set appearance.branchLineWidth=1.0;
	set appearance.branchMinLineWidth=0.0;
	set appearance.branchWidthAttribute="Fixed";
	set appearance.foregroundColour=#000000;
	set appearance.hilightingGradient=false;
	set appearance.selectionColour=#2d3680;
	set branchLabels.colorAttribute="User selection";
	set branchLabels.displayAttribute="Branch times";
	set branchLabels.fontName="Trebuchet MS";
	set branchLabels.fontSize=14;
	set branchLabels.fontStyle=0;
	set branchLabels.isShown=false;
	set branchLabels.significantDigits=4;
	set layout.expansion=0;
	set layout.layoutType="RECTILINEAR";
	set layout.zoom=0;
	set legend.attribute=null;
	set legend.fontSize=10.0;
	set legend.isShown=false;
	set legend.significantDigits=4;
	set nodeBars.barWidth=4.0;
	set nodeBars.displayAttribute=null;
	set nodeBars.isShown=false;
	set nodeLabels.colorAttribute="User selection";
	set nodeLabels.displayAttribute="Bootstrap";
	set nodeLabels.fontName="Trebuchet MS";
	set nodeLabels.fontSize=14;
	set nodeLabels.fontStyle=0;
	set nodeLabels.isShown=true;
	set nodeLabels.significantDigits=4;
	set nodeShapeExternal.colourAttribute=null;
	set nodeShapeExternal.isShown=false;
	set nodeShapeExternal.minSize=10.0;
	set nodeShapeExternal.scaleType=Width;
```

```
	set nodeShapeExternal.shapeType=Circle;
	set nodeShapeExternal.size=4.0;
	set nodeShapeExternal.sizeAttribute=null;
	set nodeShapeInternal.colourAttribute=null;
	set nodeShapeInternal.isShown=false;
	set nodeShapeInternal.minSize=10.0;
	set nodeShapeInternal.scaleType=Width;
	set nodeShapeInternal.shapeType=Circle;
	set nodeShapeInternal.size=4.0;
	set nodeShapeInternal.sizeAttribute=null;
	set polarLayout.alignTipLabels=false;
	set polarLayout.angularRange=0;
	set polarLayout.rootAngle=0;
	set polarLayout.rootLength=100;
	set polarLayout.showRoot=true;
	set radialLayout.spread=0.0;
	set rectilinearLayout.alignTipLabels=false;
	set rectilinearLayout.curvature=0;
	set rectilinearLayout.rootLength=100;
	set scale.offsetAge=0.0;
	set scale.rootAge=1.0;
	set scale.scaleFactor=1.0;
	set scale.scaleRoot=false;
	set scaleAxis.automaticScale=true;
	set scaleAxis.fontSize=8.0;
	set scaleAxis.isShown=false;
	set scaleAxis.lineWidth=1.0;
	set scaleAxis.majorTicks=1.0;
	set scaleAxis.minorTicks=0.5;
	set scaleAxis.origin=0.0;
	set scaleAxis.reverseAxis=false;
	set scaleAxis.showGrid=true;
	set scaleBar.automaticScale=true;
	set scaleBar.fontSize=14.0;
	set scaleBar.isShown=true;
	set scaleBar.lineWidth=1.0;
	set scaleBar.scaleRange=0.0;
	set tipLabels.colorAttribute="User selection";
	set tipLabels.displayAttribute="Names";
	set tipLabels.fontName="Trebuchet MS";
	set tipLabels.fontSize=14;
	set tipLabels.fontStyle=0;
	set tipLabels.isShown=true;
	set tipLabels.significantDigits=4;
	set trees.order=false;
	set trees.orderType="increasing";
	set trees.rooting=true;
	set trees.rootingType="User Selection";
	set trees.transform=false;
	set trees.transformType="cladogram";
end;
```

```
#NEXUS
begin taxa;
	dimensions ntax=10;
	taxlabels
	LCN15-1_Dnov
	LCN15-1_Ecab
	LCN15-1_Ggal
	LCN15-1_Lafr
	LCN15-2_Dnov
	LCN15-2_Ecab
	LCN15-2_Ggal
	LCN15-2_Lafr
	LCN15_Hsap
	LCN15_Shar
;
end;

begin trees;
	tree tree_1 = [&R] (LCN15-1_Dnov:0.260182,LCN15-1_Ggal:0.490986,(((LCN15-2_Dnov:0.252821,LCN15-2_Ggal:0.369546)[&Bootstrap=99,!rotate=true]:0.386966,(LCN15_Shar:0.264951,((LCN15-2_Ecab:0.117046,LCN15_Hsap:0.116043)[&Bootstrap=51]:0.02419,LCN15-2_Lafr:0.090936)[&Bootstrap=68]:0.086507)[&Bootstrap=100]:0.527443)[&Bootstrap=77,!rotate=false]:0.158681,(LCN15-1_Ecab:0.38408,LCN15-1_Lafr:0.050266)[&Bootstrap=100]:0.731664)[&Bootstrap=100,!rotate=false]:0.698874);
end;

begin figtree;
	set appearance.backgroundColorAttribute="Default";
	set appearance.backgroundColour=#ffffff;
	set appearance.branchColorAttribute="User selection";
	set appearance.branchColorGradient=false;
	set appearance.branchLineWidth=1.0;
	set appearance.branchMinLineWidth=0.0;
	set appearance.branchWidthAttribute="Fixed";
	set appearance.foregroundColour=#000000;
	set appearance.hilightingGradient=false;
	set appearance.selectionColour=#2d3680;
	set branchLabels.colorAttribute="User selection";
	set branchLabels.displayAttribute="Branch times";
	set branchLabels.fontName="Trebuchet MS";
	set branchLabels.fontSize=14;
	set branchLabels.fontStyle=0;
	set branchLabels.isShown=false;
	set branchLabels.significantDigits=4;
	set layout.expansion=0;
	set layout.layoutType="RECTILINEAR";
	set layout.zoom=0;
	set legend.attribute=null;
	set legend.fontSize=10.0;
	set legend.isShown=false;
	set legend.significantDigits=4;
	set nodeBars.barWidth=4.0;
	set nodeBars.displayAttribute=null;
	set nodeBars.isShown=false;
	set nodeLabels.colorAttribute="User selection";
	set nodeLabels.displayAttribute="Bootstrap";
	set nodeLabels.fontName="Trebuchet MS";
	set nodeLabels.fontSize=14;
```

set nodeLabels.fontStyle=0;
set nodeLabels.isShown=true;
set nodeLabels.significantDigits=4;
set nodeShapeExternal.colourAttribute=null;
set nodeShapeExternal.isShown=false;
set nodeShapeExternal.minSize=10.0;
set nodeShapeExternal.scaleType=Width;
set nodeShapeExternal.shapeType=Circle;
set nodeShapeExternal.size=4.0;
set nodeShapeExternal.sizeAttribute=null;
set nodeShapeInternal.colourAttribute=null;
set nodeShapeInternal.isShown=false;
set nodeShapeInternal.minSize=10.0;
set nodeShapeInternal.scaleType=Width;
set nodeShapeInternal.shapeType=Circle;
set nodeShapeInternal.size=4.0;
set nodeShapeInternal.sizeAttribute=null;
set polarLayout.alignTipLabels=false;
set polarLayout.angularRange=0;
set polarLayout.rootAngle=0;
set polarLayout.rootLength=100;
set polarLayout.showRoot=true;
set radialLayout.spread=0.0;
set rectilinearLayout.alignTipLabels=false;
set rectilinearLayout.curvature=0;
set rectilinearLayout.rootLength=100;
set scale.offsetAge=0.0;
set scale.rootAge=1.0;
set scale.scaleFactor=1.0;
set scale.scaleRoot=false;
set scaleAxis.automaticScale=true;
set scaleAxis.fontSize=8.0;
set scaleAxis.isShown=false;
set scaleAxis.lineWidth=1.0;
set scaleAxis.majorTicks=1.0;
set scaleAxis.minorTicks=0.5;
set scaleAxis.origin=0.0;
set scaleAxis.reverseAxis=false;
set scaleAxis.showGrid=true;
set scaleBar.automaticScale=true;
set scaleBar.fontSize=14.0;
set scaleBar.isShown=true;
set scaleBar.lineWidth=1.0;
set scaleBar.scaleRange=0.0;
set tipLabels.colorAttribute="User selection";
set tipLabels.displayAttribute="Names";
set tipLabels.fontName="Trebuchet MS";
set tipLabels.fontSize=14;
set tipLabels.fontStyle=0;
set tipLabels.isShown=true;
set tipLabels.significantDigits=4;
set trees.order=true;
set trees.orderType="increasing";
set trees.rooting=true;
set trees.rootingType="User Selection";
set trees.transform=false;
set trees.transformType="cladogram";

```
end;
```

```
#NEXUS
begin taxa;
	dimensions ntax=14;
	taxlabels
	ORM-1_Ecab
	ORM-1_Lafr
	ORM-1a_Mmus
	ORM-1b_Mmus
	ORM-1c_Mmus
	ORM-2_Ecab
	ORM-2_Lafr
	ORM-3_Ecab
	ORM-4_Ecab
	ORM1_Hsap
	ORM2_Hsap
	ORM_Dnov
	ORM_Ggal
	ORM_Shar
;
end;

begin trees;
	tree tree_1 = [&R] (ORM_Dnov[&!rotate=false]:0.270531,ORM_Ggal:0.283524,(ORM_Shar:0.726228,
((((ORM-4_Ecab:0.419886,ORM-1_Ecab:0.161821)[&Bootstrap=61]:0.044743,(ORM-2_Ecab:0.122564,ORM-
3_Ecab:0.190499)[&Bootstrap=82,!rotate=true]:0.099845)[&Bootstrap=98]:0.229287,
((ORM2_Hsap:0.049736,ORM1_Hsap:0.074187)[&Bootstrap=100]:0.221859,((ORM-1c_Mmus:0.170148,ORM-
1b_Mmus:0.068798)[&Bootstrap=91]:0.064446,ORM-1a_Mmus:0.071041)[&Bootstrap=100]:0.595439)
[&Bootstrap=52,!rotate=true]:0.055736)[&Bootstrap=53,!rotate=true]:0.112814,(ORM-1_Lafr:0.110769,ORM-
2_Lafr:0.121852)[&Bootstrap=75]:0.080356)[&Bootstrap=74]:0.389695)[&Bootstrap=100,!rotate=true]:1.34001)
[&!rotate=true];
end;

begin figtree;
	set appearance.backgroundColorAttribute="Default";
	set appearance.backgroundColour=#ffffff;
	set appearance.branchColorAttribute="User selection";
	set appearance.branchColorGradient=false;
	set appearance.branchLineWidth=1.0;
	set appearance.branchMinLineWidth=0.0;
	set appearance.branchWidthAttribute="Fixed";
	set appearance.foregroundColour=#000000;
	set appearance.hilightingGradient=false;
	set appearance.selectionColour=#2d3680;
	set branchLabels.colorAttribute="User selection";
	set branchLabels.displayAttribute="Branch times";
	set branchLabels.fontName="Adobe Devanagari";
	set branchLabels.fontSize=8;
	set branchLabels.fontStyle=0;
	set branchLabels.isShown=false;
	set branchLabels.significantDigits=4;
	set layout.expansion=0;
	set layout.layoutType="RECTILINEAR";
	set layout.zoom=0;
	set legend.attribute=null;
	set legend.fontSize=10.0;
	set legend.isShown=false;
	set legend.significantDigits=4;
```

```
set nodeBars.barWidth=4.0;
set nodeBars.displayAttribute=null;
set nodeBars.isShown=false;
set nodeLabels.colorAttribute="User selection";
set nodeLabels.displayAttribute="Bootstrap";
set nodeLabels.fontName="Trebuchet MS";
set nodeLabels.fontSize=14;
set nodeLabels.fontStyle=0;
set nodeLabels.isShown=true;
set nodeLabels.significantDigits=4;
set nodeShapeExternal.colourAttribute=null;
set nodeShapeExternal.isShown=false;
set nodeShapeExternal.minSize=10.0;
set nodeShapeExternal.scaleType=Width;
set nodeShapeExternal.shapeType=Circle;
set nodeShapeExternal.size=4.0;
set nodeShapeExternal.sizeAttribute=null;
set nodeShapeInternal.colourAttribute=null;
set nodeShapeInternal.isShown=false;
set nodeShapeInternal.minSize=10.0;
set nodeShapeInternal.scaleType=Width;
set nodeShapeInternal.shapeType=Circle;
set nodeShapeInternal.size=4.0;
set nodeShapeInternal.sizeAttribute=null;
set polarLayout.alignTipLabels=false;
set polarLayout.angularRange=0;
set polarLayout.rootAngle=0;
set polarLayout.rootLength=100;
set polarLayout.showRoot=true;
set radialLayout.spread=0.0;
set rectilinearLayout.alignTipLabels=false;
set rectilinearLayout.curvature=0;
set rectilinearLayout.rootLength=100;
set scale.offsetAge=0.0;
set scale.rootAge=1.0;
set scale.scaleFactor=1.0;
set scale.scaleRoot=false;
set scaleAxis.automaticScale=true;
set scaleAxis.fontSize=8.0;
set scaleAxis.isShown=false;
set scaleAxis.lineWidth=1.0;
set scaleAxis.majorTicks=1.0;
set scaleAxis.minorTicks=0.5;
set scaleAxis.origin=0.0;
set scaleAxis.reverseAxis=false;
set scaleAxis.showGrid=true;
set scaleBar.automaticScale=true;
set scaleBar.fontSize=10.0;
set scaleBar.isShown=true;
set scaleBar.lineWidth=1.0;
set scaleBar.scaleRange=0.0;
set tipLabels.colorAttribute="User selection";
set tipLabels.displayAttribute="Names";
set tipLabels.fontName="Trebuchet MS";
set tipLabels.fontSize=14;
set tipLabels.fontStyle=0;
set tipLabels.isShown=true;
```

```
	set tipLabels.significantDigits=4;
	set trees.order=false;
	set trees.orderType="increasing";
	set trees.rooting=true;
	set trees.rootingType="User Selection";
	set trees.transform=false;
	set trees.transformType="cladogram";
end;
```

```
#NEXUS
begin taxa;
	dimensions ntax=6;
	taxlabels
	PAEP-1a_Ecab
	PAEP-1b_Ecab
	PAEP-like_Shar
	PAEP_Hsap
	PAEP_Lafr
	PAEP_Shar
;
end;

begin trees;
	tree tree_1 = [&R] (PAEP-like_Shar:1.884851,PAEP_Shar:1.081065,((PAEP-1b_Ecab:0.096565,(PAEP-
1a_Ecab:0.134906,PAEP_Lafr:0.526261)[&Bootstrap=50,!rotate=true]:0.211147)
[&Bootstrap=26]:0.136195,PAEP_Hsap:0.506513)[&Bootstrap=71,!rotate=false]:0.481654);
end;

begin figtree;
	set appearance.backgroundColorAttribute="Default";
	set appearance.backgroundColour=#ffffff;
	set appearance.branchColorAttribute="User selection";
	set appearance.branchColorGradient=false;
	set appearance.branchLineWidth=1.0;
	set appearance.branchMinLineWidth=0.0;
	set appearance.branchWidthAttribute="Fixed";
	set appearance.foregroundColour=#000000;
	set appearance.hilightingGradient=false;
	set appearance.selectionColour=#2d3680;
	set branchLabels.colorAttribute="User selection";
	set branchLabels.displayAttribute="Branch times";
	set branchLabels.fontName="Trebuchet MS";
	set branchLabels.fontSize=14;
	set branchLabels.fontStyle=0;
	set branchLabels.isShown=false;
	set branchLabels.significantDigits=4;
	set layout.expansion=0;
	set layout.layoutType="RECTILINEAR";
	set layout.zoom=0;
	set legend.attribute=null;
	set legend.fontSize=10.0;
	set legend.isShown=false;
	set legend.significantDigits=4;
	set nodeBars.barWidth=4.0;
	set nodeBars.displayAttribute=null;
	set nodeBars.isShown=false;
	set nodeLabels.colorAttribute="User selection";
	set nodeLabels.displayAttribute="Bootstrap";
	set nodeLabels.fontName="Trebuchet MS";
	set nodeLabels.fontSize=14;
	set nodeLabels.fontStyle=0;
	set nodeLabels.isShown=true;
	set nodeLabels.significantDigits=4;
	set nodeShapeExternal.colourAttribute=null;
	set nodeShapeExternal.isShown=false;
	set nodeShapeExternal.minSize=10.0;
```

```
	set nodeShapeExternal.scaleType=Width;
	set nodeShapeExternal.shapeType=Circle;
	set nodeShapeExternal.size=4.0;
	set nodeShapeExternal.sizeAttribute=null;
	set nodeShapeInternal.colourAttribute=null;
	set nodeShapeInternal.isShown=false;
	set nodeShapeInternal.minSize=10.0;
	set nodeShapeInternal.scaleType=Width;
	set nodeShapeInternal.shapeType=Circle;
	set nodeShapeInternal.size=4.0;
	set nodeShapeInternal.sizeAttribute=null;
	set polarLayout.alignTipLabels=false;
	set polarLayout.angularRange=0;
	set polarLayout.rootAngle=0;
	set polarLayout.rootLength=100;
	set polarLayout.showRoot=true;
	set radialLayout.spread=0.0;
	set rectilinearLayout.alignTipLabels=false;
	set rectilinearLayout.curvature=0;
	set rectilinearLayout.rootLength=100;
	set scale.offsetAge=0.0;
	set scale.rootAge=1.0;
	set scale.scaleFactor=1.0;
	set scale.scaleRoot=false;
	set scaleAxis.automaticScale=true;
	set scaleAxis.fontSize=8.0;
	set scaleAxis.isShown=false;
	set scaleAxis.lineWidth=1.0;
	set scaleAxis.majorTicks=1.0;
	set scaleAxis.minorTicks=0.5;
	set scaleAxis.origin=0.0;
	set scaleAxis.reverseAxis=false;
	set scaleAxis.showGrid=true;
	set scaleBar.automaticScale=true;
	set scaleBar.fontSize=14.0;
	set scaleBar.isShown=true;
	set scaleBar.lineWidth=1.0;
	set scaleBar.scaleRange=0.0;
	set tipLabels.colorAttribute="User selection";
	set tipLabels.displayAttribute="Names";
	set tipLabels.fontName="Trebuchet MS";
	set tipLabels.fontSize=14;
	set tipLabels.fontStyle=0;
	set tipLabels.isShown=true;
	set tipLabels.significantDigits=4;
	set trees.order=true;
	set trees.orderType="increasing";
	set trees.rooting=true;
	set trees.rootingType="User Selection";
	set trees.transform=false;
	set trees.transformType="cladogram";
end;
```

```
#NEXUS
begin taxa;
	dimensions ntax=40;
	taxlabels
	LCN12_Ecab
	LCN12_Hsap
	LCN12_Lafr
	LCN12_Mmus
	LCN2_Ecab
	LCN2_Hsap
	LCN2_Lafr
	LCN2_Mmus
	LCN2_Shar
	PTGDS-1_Hcom
	PTGDS-1_Saur
	PTGDS-1_Smax
	PTGDS-1_Ssal
	PTGDS-1a_Drer
	PTGDS-1b_Drer
	PTGDS-2_Drer
	PTGDS-2_Hcom
	PTGDS-2_Saur
	PTGDS-2_Smax
	PTGDS-2a_Ssal
	PTGDS-2b_Ssal
	PTGDS-2c_Ssal
	PTGDS-3_Hcom
	PTGDS-3_Saur
	PTGDS-3_Smax
	PTGDS-3a_Drer
	PTGDS-3a_Ssal
	PTGDS-3b_Drer
	PTGDS-3b_Ssal
	PTGDS_Cmil
	PTGDS_Cpor
	PTGDS_Dnov
	PTGDS_Ecab
	PTGDS_Ggal
	PTGDS_Hsap
	PTGDS_Lafr
	PTGDS_Lcha
	PTGDS_Locu
	PTGDS_Mmus
	PTGDS_Shar
;
end;

begin trees;
	tree tree_1 = [&R] (PTGDS-1a_Drer:0.042283,PTGDS-1b_Drer:0.213513,(((PTGDS-2_Drer:0.291773,
(((PTGDS-2_Smax:0.147517,PTGDS-2_Saur:0.08886)[&Bootstrap=99]:0.039161,PTGDS-2_Hcom:0.218502)
[&Bootstrap=99]:0.057051,((PTGDS-2b_Ssal:0.064726,PTGDS-2c_Ssal:0.585429)
[&Bootstrap=99,!rotate=true]:0.052783,PTGDS-2a_Ssal:0.067509)[&Bootstrap=97]:0.074272)
[&Bootstrap=92]:0.047529)[&Bootstrap=89]:0.071783,((PTGDS_Cmil[&!rotate=true]:0.571225,
(((PTGDS_Cpor:0.148234,(PTGDS_Dnov:0.036813,PTGDS_Ggal:0.187463)
[&Bootstrap=100,!rotate=true]:0.175403)[&Bootstrap=97]:0.107873,((PTGDS_Shar:0.33868,
((PTGDS_Ecab:0.232594,(PTGDS_Hsap:0.08364,PTGDS_Mmus:0.225487)
[&Bootstrap=56,!rotate=true]:0.037866)[&Bootstrap=52]:0.039706,PTGDS_Lafr:0.109769)
```

```
[&Bootstrap=100]:0.334521)[&Bootstrap=75]:0.158617,((((((LCN2_Shar:0.591446,LCN2_Mmus:0.246142)
[&Bootstrap=46]:0.068399,LCN2_Hsap:0.26309)[&Bootstrap=41]:0.050268,LCN2_Lafr:0.15545)
[&Bootstrap=58]:0.079498,LCN2_Ecab:0.110943)[&Bootstrap=82]:0.233483,LCN12_Lafr:0.927337)
[&Bootstrap=82]:0.136053,(LCN12_Ecab:0.362979,(LCN12_Hsap:0.23819,LCN12_Mmus:0.454813)
[&Bootstrap=46,!rotate=true]:0.034604)[&Bootstrap=100]:0.741986)[&Bootstrap=100]:0.767986)
[&Bootstrap=92]:0.181698)[&Bootstrap=99]:0.324528,PTGDS_Lcha:0.566882)[&Bootstrap=91]:0.182154)
[&Bootstrap=67]:0.058496,(((PTGDS-3b_Drer:0.741034,PTGDS-3a_Drer:0.337812)[&Bootstrap=65]:0.104325,
((((PTGDS-3_Smax:0.132514,PTGDS-3_Saur:0.20665)[&Bootstrap=97,!rotate=true]:0.057176,PTGDS-
3_Hcom:0.072963)[&Bootstrap=99]:0.105374,PTGDS-3b_Ssal:0.966475)[&Bootstrap=85]:0.078141,PTGDS-
3a_Ssal:0.116887)[&Bootstrap=80]:0.105152)[&Bootstrap=83]:0.098599,PTGDS_Locu:0.143098)
[&Bootstrap=100]:0.785296)[&Bootstrap=100]:0.511427)[&Bootstrap=89]:0.102199,(((PTGDS-
1_Smax:0.185575,PTGDS-1_Saur:0.136085)[&Bootstrap=97]:0.032878,PTGDS-1_Hcom:0.34009)
[&Bootstrap=80]:0.045054,PTGDS-1_Ssal:0.221963)[&Bootstrap=80]:0.076558)
[&Bootstrap=100,!rotate=true]:0.178576);
end;

begin figtree;
	set appearance.backgroundColorAttribute="Default";
	set appearance.backgroundColour=#ffffff;
	set appearance.branchColorAttribute="User selection";
	set appearance.branchColorGradient=false;
	set appearance.branchLineWidth=1.0;
	set appearance.branchMinLineWidth=0.0;
	set appearance.branchWidthAttribute="Fixed";
	set appearance.foregroundColour=#000000;
	set appearance.hilightingGradient=false;
	set appearance.selectionColour=#2d3680;
	set branchLabels.colorAttribute="User selection";
	set branchLabels.displayAttribute="Branch times";
	set branchLabels.fontName="Adobe Devanagari";
	set branchLabels.fontSize=8;
	set branchLabels.fontStyle=0;
	set branchLabels.isShown=false;
	set branchLabels.significantDigits=4;
	set layout.expansion=0;
	set layout.layoutType="RECTILINEAR";
	set layout.zoom=0;
	set legend.attribute=null;
	set legend.fontSize=10.0;
	set legend.isShown=false;
	set legend.significantDigits=4;
	set nodeBars.barWidth=4.0;
	set nodeBars.displayAttribute=null;
	set nodeBars.isShown=false;
	set nodeLabels.colorAttribute="User selection";
	set nodeLabels.displayAttribute="Bootstrap";
	set nodeLabels.fontName="Trebuchet MS";
	set nodeLabels.fontSize=14;
	set nodeLabels.fontStyle=0;
	set nodeLabels.isShown=true;
	set nodeLabels.significantDigits=4;
	set nodeShapeExternal.colourAttribute=null;
	set nodeShapeExternal.isShown=false;
	set nodeShapeExternal.minSize=10.0;
	set nodeShapeExternal.scaleType=Width;
	set nodeShapeExternal.shapeType=Circle;
	set nodeShapeExternal.size=4.0;
```

```
	set nodeShapeExternal.sizeAttribute=null;
	set nodeShapeInternal.colourAttribute=null;
	set nodeShapeInternal.isShown=false;
	set nodeShapeInternal.minSize=10.0;
	set nodeShapeInternal.scaleType=Width;
	set nodeShapeInternal.shapeType=Circle;
	set nodeShapeInternal.size=4.0;
	set nodeShapeInternal.sizeAttribute=null;
	set polarLayout.alignTipLabels=false;
	set polarLayout.angularRange=0;
	set polarLayout.rootAngle=0;
	set polarLayout.rootLength=100;
	set polarLayout.showRoot=true;
	set radialLayout.spread=0.0;
	set rectilinearLayout.alignTipLabels=false;
	set rectilinearLayout.curvature=0;
	set rectilinearLayout.rootLength=100;
	set scale.offsetAge=0.0;
	set scale.rootAge=1.0;
	set scale.scaleFactor=1.0;
	set scale.scaleRoot=false;
	set scaleAxis.automaticScale=true;
	set scaleAxis.fontSize=8.0;
	set scaleAxis.isShown=false;
	set scaleAxis.lineWidth=1.0;
	set scaleAxis.majorTicks=1.0;
	set scaleAxis.minorTicks=0.5;
	set scaleAxis.origin=0.0;
	set scaleAxis.reverseAxis=false;
	set scaleAxis.showGrid=true;
	set scaleBar.automaticScale=true;
	set scaleBar.fontSize=10.0;
	set scaleBar.isShown=true;
	set scaleBar.lineWidth=1.0;
	set scaleBar.scaleRange=0.0;
	set tipLabels.colorAttribute="User selection";
	set tipLabels.displayAttribute="Names";
	set tipLabels.fontName="Trebuchet MS";
	set tipLabels.fontSize=14;
	set tipLabels.fontStyle=0;
	set tipLabels.isShown=true;
	set tipLabels.significantDigits=4;
	set trees.order=true;
	set trees.orderType="increasing";
	set trees.rooting=true;
	set trees.rootingType="User Selection";
	set trees.transform=false;
	set trees.transformType="cladogram";
end;
```

```
#NEXUS
begin taxa;
	dimensions ntax=33;
	taxlabels
	RBP4-1_Cpor
	RBP4-1_Dnov
	RBP4-1_Drer
	RBP4-1_Ggal
	RBP4-1_Hcom
	RBP4-1_Lcha
	RBP4-1_Locu
	RBP4-1_Saur
	RBP4-1_Shar
	RBP4-1_Smax
	RBP4-1a_Ssal
	RBP4-1b_Ssal
	RBP4-2_Cpor
	RBP4-2_Dnov
	RBP4-2_Drer
	RBP4-2_Ggal
	RBP4-2_Lcha
	RBP4-2_Locu
	RBP4-2_Shar
	RBP4-2a_Hcom
	RBP4-2a_Saur
	RBP4-2a_Smax
	RBP4-2a_Ssal
	RBP4-2b_Hcom
	RBP4-2b_Saur
	RBP4-2b_Smax
	RBP4-2b_Ssal
	RBP4-2c_Ssal
	RBP4_Cmil
	RBP4_Ecab
	RBP4_Hsap
	RBP4_Lafr
	RBP4_Mmus
;
end;

begin trees;
	tree tree_1 = [&R] (RBP4-2_Drer:0.085732,((((RBP4-2a_Smax:0.048548,RBP4-2a_Hcom:0.094217)
[&Bootstrap=88,!rotate=true]:0.012967,RBP4-2a_Saur:0.035033)[&Bootstrap=89]:0.033496,((RBP4-
2c_Ssal:0.021962,RBP4-2b_Ssal:0.021191)[&Bootstrap=100]:0.091744,(RBP4-2b_Saur:0.103195,(RBP4-
2b_Hcom:0.04926,RBP4-2b_Smax:0.117011)[&Bootstrap=99,!rotate=true]:0.05643)[&Bootstrap=94]:0.048584)
[&Bootstrap=79]:0.045709)[&Bootstrap=67]:0.009666,RBP4-2a_Ssal:0.050649)[&Bootstrap=75]:0.019184,(RBP4-
2_Locu:0.072623,((((RBP4-2_Dnov:0.030744,RBP4-2_Ggal:2.0E-6)[&Bootstrap=90]:0.033084,(((((RBP4-
1_Drer:0.235022,(RBP4-1a_Ssal:0.03481,RBP4-1b_Ssal:0.024218)[&Bootstrap=100]:0.135967)
[&Bootstrap=56]:0.07277,RBP4-1_Locu:0.259556)[&Bootstrap=68]:2.0E-6,((RBP4-1_Saur:0.23278,RBP4-
1_Smax:0.495969)[&Bootstrap=59,!rotate=true]:0.046958,RBP4-1_Hcom:0.489546)[&Bootstrap=64]:0.072285)
[&Bootstrap=100]:0.519445,RBP4-1_Lcha:0.69408)[&Bootstrap=82]:0.117437,((RBP4-1_Cpor:0.293119,(RBP4-
1_Dnov:0.0383,RBP4-1_Ggal:0.00213)[&Bootstrap=97]:0.0975)[&Bootstrap=92]:0.105729,
(((RBP4_Ecab:0.053207,RBP4_Lafr:0.194053)[&Bootstrap=86,!rotate=true]:0.038257,
(RBP4_Mmus:0.224272,RBP4-1_Shar:0.400361)[&Bootstrap=60,!rotate=true]:0.045192)
[&Bootstrap=49,!rotate=true]:0.00374,RBP4_Hsap:2.0E-6)[&Bootstrap=88]:0.132023)[&Bootstrap=87]:0.14487)
[&Bootstrap=100,!rotate=true]:1.439956)[&Bootstrap=34]:3.0E-6,((RBP4-2_Cpor:0.226846,RBP4-
2_Shar:0.277081)[&Bootstrap=93]:0.067957,RBP4-2_Lcha:0.281293)[&Bootstrap=61]:0.060378)
```

```
[&Bootstrap=57]:0.060177,RBP4_Cmil:0.314171)[&Bootstrap=92]:0.118542)[&Bootstrap=79]:0.076496);
end;

begin figtree;
	set appearance.backgroundColorAttribute="Default";
	set appearance.backgroundColour=#ffffff;
	set appearance.branchColorAttribute="User selection";
	set appearance.branchColorGradient=false;
	set appearance.branchLineWidth=1.0;
	set appearance.branchMinLineWidth=0.0;
	set appearance.branchWidthAttribute="Fixed";
	set appearance.foregroundColour=#000000;
	set appearance.hilightingGradient=false;
	set appearance.selectionColour=#2d3680;
	set branchLabels.colorAttribute="User selection";
	set branchLabels.displayAttribute="Branch times";
	set branchLabels.fontName="Adobe Devanagari";
	set branchLabels.fontSize=8;
	set branchLabels.fontStyle=0;
	set branchLabels.isShown=false;
	set branchLabels.significantDigits=4;
	set layout.expansion=0;
	set layout.layoutType="RECTILINEAR";
	set layout.zoom=0;
	set legend.attribute="Bootstrap";
	set legend.fontSize=10.0;
	set legend.isShown=false;
	set legend.significantDigits=4;
	set nodeBars.barWidth=4.0;
	set nodeBars.displayAttribute=null;
	set nodeBars.isShown=false;
	set nodeLabels.colorAttribute="User selection";
	set nodeLabels.displayAttribute="Bootstrap";
	set nodeLabels.fontName="Trebuchet MS";
	set nodeLabels.fontSize=14;
	set nodeLabels.fontStyle=0;
	set nodeLabels.isShown=false;
	set nodeLabels.significantDigits=4;
	set nodeShapeExternal.colourAttribute="User selection";
	set nodeShapeExternal.isShown=false;
	set nodeShapeExternal.minSize=10.0;
	set nodeShapeExternal.scaleType=Width;
	set nodeShapeExternal.shapeType=Circle;
	set nodeShapeExternal.size=4.0;
	set nodeShapeExternal.sizeAttribute="Fixed";
	set nodeShapeInternal.colourAttribute="User selection";
	set nodeShapeInternal.isShown=false;
	set nodeShapeInternal.minSize=10.0;
	set nodeShapeInternal.scaleType=Width;
	set nodeShapeInternal.shapeType=Circle;
	set nodeShapeInternal.size=4.0;
	set nodeShapeInternal.sizeAttribute="Fixed";
	set polarLayout.alignTipLabels=false;
	set polarLayout.angularRange=0;
	set polarLayout.rootAngle=0;
	set polarLayout.rootLength=100;
	set polarLayout.showRoot=true;
```

```
	set radialLayout.spread=0.0;
	set rectilinearLayout.alignTipLabels=false;
	set rectilinearLayout.curvature=0;
	set rectilinearLayout.rootLength=100;
	set scale.offsetAge=0.0;
	set scale.rootAge=1.0;
	set scale.scaleFactor=1.0;
	set scale.scaleRoot=false;
	set scaleAxis.automaticScale=true;
	set scaleAxis.fontSize=8.0;
	set scaleAxis.isShown=false;
	set scaleAxis.lineWidth=1.0;
	set scaleAxis.majorTicks=1.0;
	set scaleAxis.minorTicks=0.5;
	set scaleAxis.origin=0.0;
	set scaleAxis.reverseAxis=false;
	set scaleAxis.showGrid=true;
	set scaleBar.automaticScale=true;
	set scaleBar.fontSize=10.0;
	set scaleBar.isShown=true;
	set scaleBar.lineWidth=1.0;
	set scaleBar.scaleRange=0.0;
	set tipLabels.colorAttribute="User selection";
	set tipLabels.displayAttribute="Names";
	set tipLabels.fontName="Trebuchet MS";
	set tipLabels.fontSize=14;
	set tipLabels.fontStyle=0;
	set tipLabels.isShown=true;
	set tipLabels.significantDigits=4;
	set trees.order=true;
	set trees.orderType="increasing";
	set trees.rooting=true;
	set trees.rootingType="User Selection";
	set trees.transform=false;
	set trees.transformType="cladogram";
end;
```
