# Supplementary figures and images for: An Evolutionary Perspective of the Lipocalin Protein Family
Source: Front Physiol. 2021 Aug 23;12:718983. doi: 10.3389/fphys.2021.718983 (PMC8420045; doi:10.3389/fphys.2021.718983)

Pairwise MSA and JDet specificity-determining positions (XDet:0.6, Entropy:2.5 and S3:10.0 thresholds).

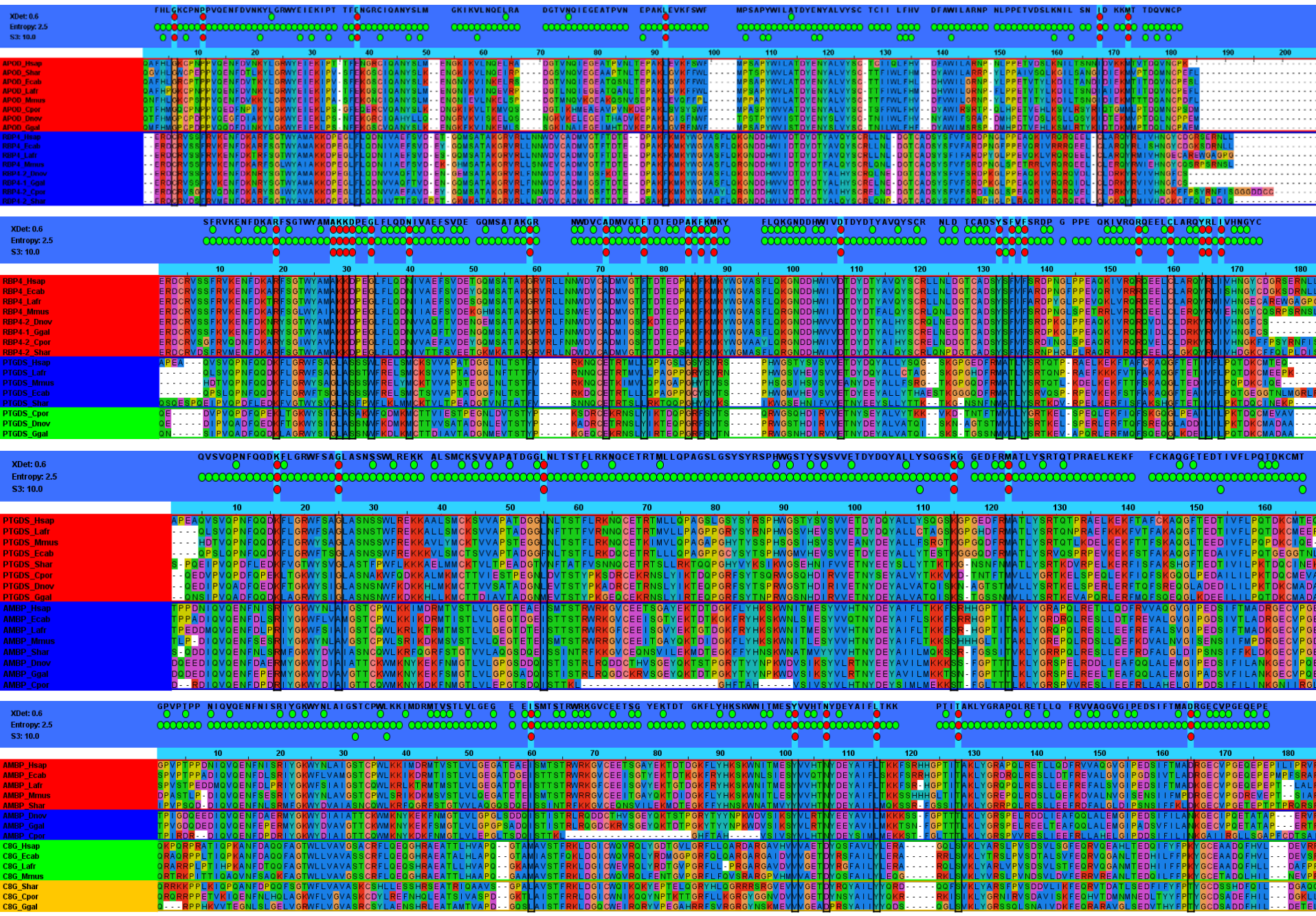

Supplement: Supplementary file 3 [file Data_Sheet_3.PDF]
